# Pre-synaptic Muscarinic Excitation Enhances the Discrimination of Looming Stimuli in a Collision-Detection Neuron

**DOI:** 10.1016/j.celrep.2018.04.079

**Published:** 2018-05-22

**Authors:** Ying Zhu, Richard B. Dewell, Hongxia Wang, Fabrizio Gabbiani

**Affiliations:** 1 Department of Neuroscience, Baylor College of Medicine, Houston, TX 77030, USA; 2 Quantitative and Computational Biosciences Graduate Program, Baylor College of Medicine, Houston, TX 77030, USA; 3 Electrical and Computer Engineering Department, Rice University, Houston, TX 77005, USA

## Abstract

Visual neurons that track objects on a collision course are often finely tuned to their target stimuli because this is critical for survival. The presynaptic neural networks converging on these neurons and their role in tuning them remain poorly understood. We took advantage of well-known characteristics of one such neuron in the grasshopper visual system to investigate the properties of its presynaptic input network. We find the structure more complex than hitherto realized. In addition to dynamic lateral inhibition used to filter out background motion, presynaptic circuits include normalizing inhibition and excitatory interactions mediated by muscarinic acetylcholine receptors. These interactions preferentially boost responses to coherently expanding visual stimuli generated by colliding objects, as opposed to spatially incoherent controls, helping to discriminate between them. Hence, in addition to active dendritic conductances within collision-detecting neurons, multiple layers of inhibitory and excitatory presynaptic connections are needed to finely tune neural circuits for collision detection.

## In Brief

In the locust visual system, Zhu et al. study the presynaptic circuitry of a collision-detecting neuron. They characterize local excitatory connections mediated by muscarinic acetylcholine receptors tuning the neuron to coherently expanding visual stimuli. Additionally, they describe a global inhibitory mechanism normalizing the excitatory inputs the cell receives.

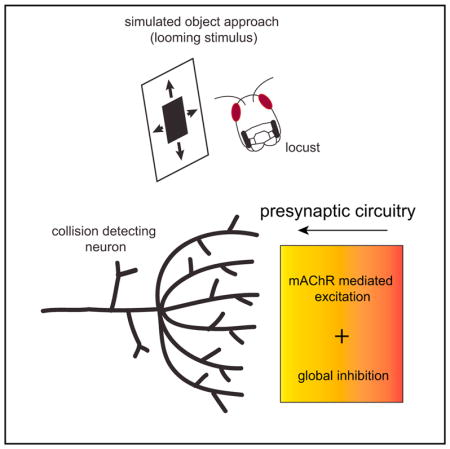

## INTRODUCTION

Both in vertebrates and invertebrates, collision-detecting neurons are typically located in higher-order nuclei and neuropils, several synapses away from the periphery. In fish, e.g., collision-detecting neurons are found in the optic tectum and the hindbrain (Preuss et al., 2006; Dunn et al., 2016; Temizer et al., 2015). In birds, they were identified in the *nucleus rotundus* of the thalamus (Sun and Frost, 1998). In mice and cats, they have been documented in the superior colliculus (Liu et al., 2011; Shang et al., 2015; Zhao et al., 2014). In insects and crustaceans, collision-detecting neurons are found in the lobula complex of the optic lobes (de Vries and Clandinin, 2012; Wu et al., 2016; Gabbiani et al., 2002; Oliva and Tomsic, 2014) and in the brain (von Reyn et al., 2014; Rind and Simmons, 1992). This leaves room for presynaptic networks to implement, through specific connectivity patterns, the neural computations required to shape their responses.

The lobula giant movement detector (LGMD) is an identified visual neuron in the third neuropil of the optic lobe of orthopteran insects such as locusts (O’Shea and Williams, 1974). It responds preferentially to objects approaching on a collision course or their two-dimensional simulations on a screen (i.e., looming stimuli; Hatsopoulos et al., 1995; Rind and Simmons, 1992) and plays a role in visually evoked escape behavior (Fotowat and Gabbiani, 2011; Santer et al., 2006). The LGMD is exquisitely tuned to looming stimuli (e.g., Judge and Rind, 1997; Gray et al., 2001; Gabbiani et al., 2001), due in part to active conductances close to its spike initiation zone and within its dendritic tree (Peron and Gabbiani, 2009; Dewell and Gabbiani, 2018). A host of neurons with response characteristics resembling those of the LGMD (Gabbiani et al., 1999) have been described (Sun and Frost, 1998; Liu et al., 2011; de Vries and Clandinin, 2012; Oliva and Tomsic, 2014; Temizer et al., 2015; Dunn et al., 2016; von Reyn et al., 2017). In none of these systems do we understand how spatio-temporal interactions within presynaptic circuits shape their responses.

The LGMD receives onto its excitatory dendritic field a retinotopic projection from an entire visual hemifield originating from ommatidia (facets) on the eye (Zhu and Gabbiani, 2016). Electron microscopy revealed that the transmedullary afferents (TmAs) building it make reciprocal synapses on their neighbors at their presynaptic terminals on the LGMD (Rind and Simmons, 1998). Histology and immunocytochemistry demonstrated that these local lateral connections are cholinergic and not GABAergic. They might thus mediate inhibition through muscarinic acetylcholine receptors (mAChRs; Rind and Simmons, 1998; Rind and Leitinger, 2000). However, muscarinic acetylcholine receptors can be excitatory (Brown, 2010). If lateral connections are inhibitory, then the activation of one afferent would inhibit adjacent ones, setting up a race between excitation and inhibition that might lead to specific responses for expanding over translating stimuli (Rind et al., 2016). By contrast, if the connections are excitatory, then the activation of one afferent would increase synaptic release from adjacent ones, enabling stronger and longer-lasting responses to coherently expanding stimuli, such as objects approaching on a collision course.

Calcium enters the LGMD excitatory dendritic field exclusively through the nicotinic receptors associated with its TmAs (Peron et al., 2009). Thus, calcium imaging accurately monitors activation of the afferents and their lateral interactions. To investigate whether lateral interactions between TmAs are excitatory or inhibitory, we locally applied the muscarinic acetylcholine receptor antagonist scopolamine or the agonist muscarine and tested responses to visual stimuli. In all cases, the lateral interactions proved to be excitatory. This raises a conundrum: because lateral connections amplify an excitatory input that grows rapidly during object approach, how is it maintained within the dynamic range of the LGMD? One possibility is for the presynaptic network to rely on global, normalizing inhibition, a feature present in many sensory circuits (Carandini and Heeger, 2011). Our evidence suggests that such inhibition is indeed present among presynaptic LGMD excitatory afferents. What role does lateral excitation play in collision detection? We show that it helps the LGMD discriminate solid objects approaching on a collision course from spatially incoherent matched controls, a computation critical to elicit appropriate escape behaviors (Dewell and Gabbiani, 2018).

## RESULTS

### Looming Stimuli Evoke an Orderly Activation of the LGMD Excitatory Dendritic Field

**Figure 1:**
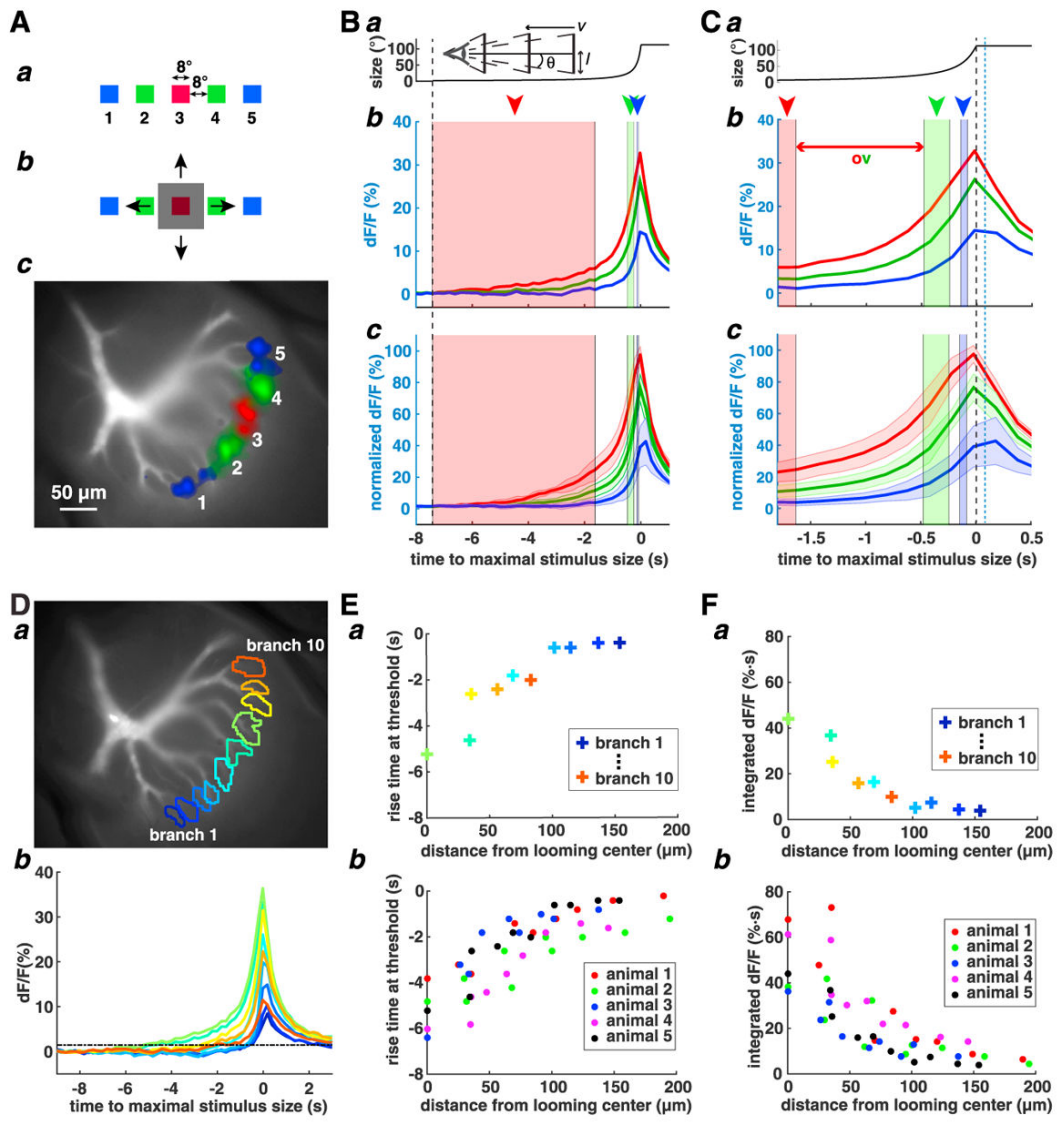
Spatio-temporal Activation of LGMD Excitatory Inputs to Looming Stimuli (A) a: to map dendritic receptive fields, brief square flashes were presented at 5 locations on the display. 3 colors represent flashes with different distances from the display center. b: a looming stimulus was then presented. It expanded from the center square and eventually covered locations where the other flashes were presented. c: LGMD excitatory dendritic field. Dendritic branches activated by the 5 flashes are shown in red, green, and blue. (B) The black dashed line indicates the start of the stimulus. a: angular size time course (*l/*|*v*| = 120 ms). Stimulus parameters were as follows: radius *l*, speed *v*, and angular size 2θ (schematics). b: average relative fluorescence change at the center branch (red) and two sets of branches symmetrically surrounding it (green and blue) in one animal. Time is relative to maximal stimulus size (80 ms before projected collision). c: mean (solid lines) and SD (shaded areas, n = 5; n, number of animals) of average dF/F (normalized to maximum dF/F in response to center square flash for each animal) at the center branch (red) and at two surrounding branch sets (green and blue). b and c: red, green, and blue rectangles (arrowheads) represent the time during which the stimulus edges moved across locations activated by red, green, and blue flashes. Over the white intermediate regions, the stimulus edge would be within the receptive fields of branches on either side. (C) The same as (B) but enlarged. The black dashed line indicates the time when the stimulus reached its maximum size; the dashed blue line represents the projected collision time. The red double arrow indicates the time interval during which the stimulus edge activates both the red and green branches (A, c) because of overlapped activation (Zhu and Gabbiani, 2016). (D) a: LGMD excitatory dendritic field with 10 selected dendritic branches indicated in colors. b: time course of average dF/F at each selected branch of a. The horizontal black dashed line is threshold for branch activation. (E) a: the y axis represents the time when the rising phase of each trace in (D), b, crosses the threshold. The branch with the earliest rise time at threshold is the looming center. The x axis represents the distance in the imaging plane from each dendritic branch to the looming center. The colors match those in (D). b: the same as a across 5 animals. All dendritic branches are plotted in the same color for each animal. (F) a: time-integrated dF/F for each dendritic branch in (D) as a function of distance from the looming center. b: the same as a across 5 animals. Data from each animal are plotted in a different color. See also Figure S1.

**Figure 2:**
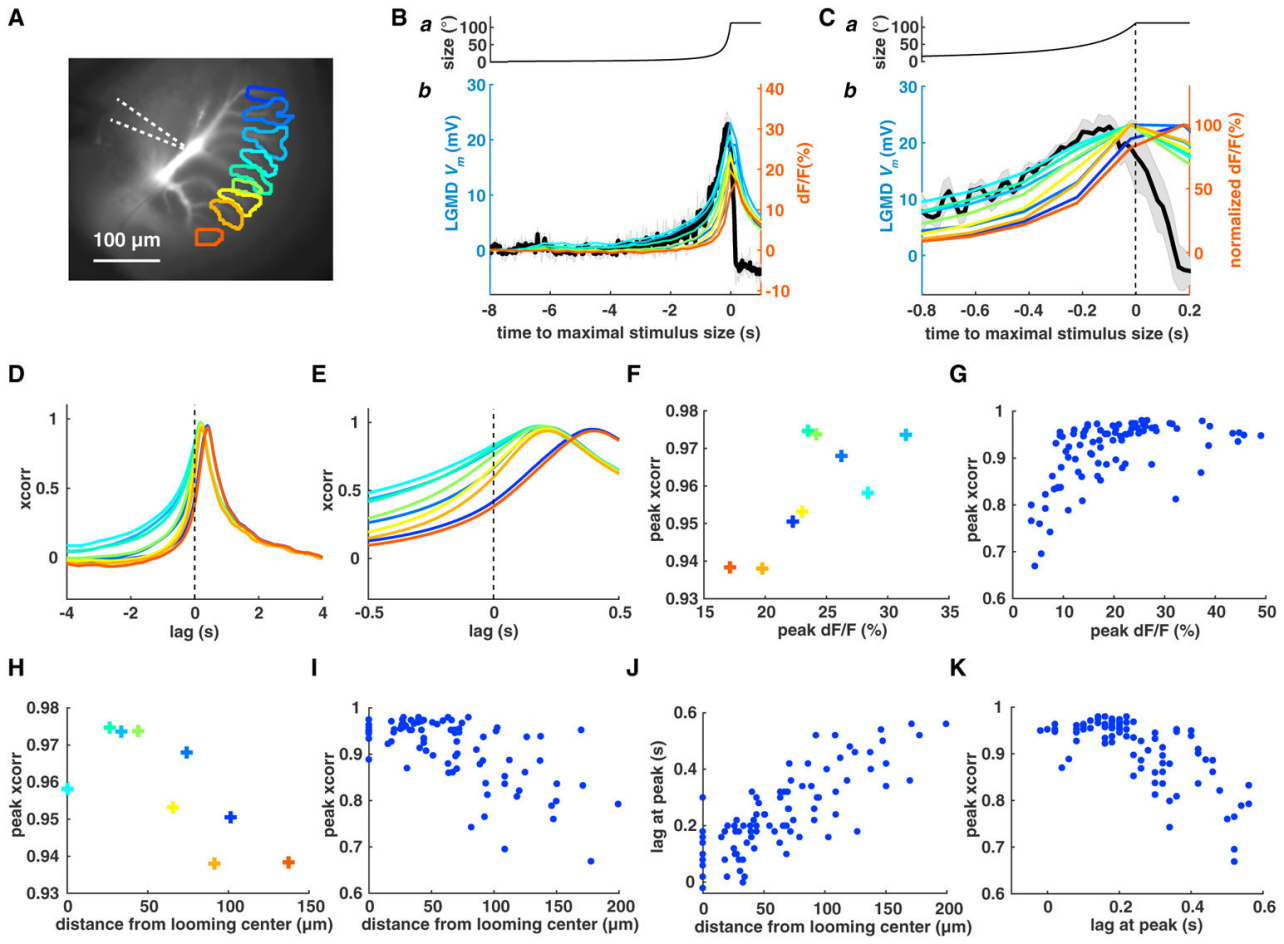
Comparison of Calcium Responses and Subthreshold *V_m_* (A) LGMD excitatory dendritic field with selected dendritic branches in different colors. White dashed lines indicate intracellular electrodes. (B) a: time course of stimulus angular size (*l/*|*v*| = 120 ms). b: LGMD dendritic membrane potential (*V_m_*; black line). Gray shading, SD (4 trials). Thin colored lines represent time courses of average dF/F within the selected dendritic branches (A). (C) Enlarged view of (B). dF/F traces were normalized to their peaks. (D) Cross-correlation (xcorr) of the *V_m_* rising phase and dF/F. The colors match (B). The black dashed line indicates zero-time lag. (E) Enlarged view of (D). (F) The peak of each cross-correlation in (D) as a function of peak dF/F for each dendritic branch in (B). (G) The same as (F) across animals. (H) The peak of each cross-correlation trace in (D) as a function of its branch distance to the looming center. (I) The same as (H) across animals. (J) Time lag at the peak of each cross-correlation trace plotted across animals as a function of its branch distance to the looming center. (K) Peak cross-correlation as a function of peak time lag across animals. In (G) and (I)–(K), n = 7, *V_m_* was recorded at 11 locations. See also Figure S2.

We started by mapping the activation pattern elicited by different regions of the visual display on the LGMD’s excitatory dendritic field (Experimental Procedures). We flashed squares at 5 locations (Figures 1Aa and S1A) and determined the activated dendritic branches (Figure 1Ac). Previous work showed that a dendritic branch is locally activated by more than one ommatidium and that overlap in dendritic activation decreases with inter-ommatidial distance, becoming close to 0 for a separation of 4 ommatidia (Zhu and Gabbiani, 2016). Because each ommatidium is receptive to ~2° of visual space (Wilson, 1975), we separated the stimuli by 8° to minimize overlap. We found that the branches activated by these stimuli intersected minimally (Figure 1Ac). How does a looming stimulus activate such a retinotopic mapping? To address this question, we presented looming stimuli with a high half-size (*l*) to speed (|*v*|) ratio and, thus, a long time of approach (Fotowat and Gabbiani, 2007), maximizing our ability to resolve temporally the associated calcium signals (Figure 1Ba; Video S1). These stimuli expanded symmetrically from the center of square 3 (Figure 1Ab). Figures 1Bb and 1Cb show the average dendritic calcium responses to these stimuli at the center branches and two sets of branches symmetrically surrounding them. Unexpectedly, given the retinotopic organization, we found that the relative fluorescence change, dF/F, of the center branches reached its peak after the edge of the looming stimulus had moved outside of the square to which they mapped. The calcium signal continued to increase even after the edges of the stimulus had expanded beyond the 4 lateral squares. The central branches also activated earlier and reached a higher peak dF/F. These results were consistent across animals (Figures 1B and 1Cc). Finally, the dF/F peak times of the center and the two sets of surrounding branches were staggered in time (Figure S1B), a feature better resolved in the next set of experiments (Figure 2J).

We analyzed the calcium responses on a finer spatial scale by considering each imaged dendritic branch (Figure 1Da). We defined a threshold activation as 5 times the dF/F baseline noise (Figure 1Db; Experimental Procedures). We call the time at which dF/F crosses this threshold relative to that at which the stimulus reaches its maximal size the rise time. We found that the branch that had the earliest rise time mapped to the center square on the display (Figures 1D and 1Ea; cf. Figure 1Ac). We call this branch the “looming center.” As predicted from retinotopy, the rise time increased with branch distance from the looming center (Figure 1Eb). dF/F integrated over time up to its peak, a measure of cumulative calcium influx, decreased as a branch lay farther from the looming center (Figure 1F). Thus, looming stimuli generate an orderly activation of dendrites that lasts over the entire stimulus and decreases in strength.

### Calcium Responses at the Looming Center Track Subthreshold Membrane Potential

dF/F changes are slower than membrane potential (*V_m_*) changes because of buffering and the binding kinetics of calcium dyes. We compared the dF/F to looming stimuli obtained using either Oregon green 1,2-Bis(2-Aminophenoxy)ethane-N,N,N′,N′-tetraacetic acid (BAPTA)-1 (OGB-1) or Oregon green BAPTA-5N (OGB-5N) and found OGB-5N to have faster kinetics (Figures S2A and S2B). We checked how closely OGB-5N calcium responses track the subthreshold membrane potential by simultaneous recordings. The calcium signals from individual dendritic branches (Figure 2A) are plotted with the median filtered *V_m_* in Figures 2B and 2C. The dF/F at the looming center best matched the subthreshold *V_m_*. This was also true at faster approach speeds (Figures S2C–S2F). The match was close over the rising phase of *V_m_* up to its peak, when synaptic excitation generating the calcium influx dominates, but not over *V_m_*’s decaying phase, when synaptic inhibition dominates (Gabbiani et al., 2002, 2005). We computed the cross-correlation (xcorr) between *V_m_* and dF/F during the rising phase of *V_m_* as a function of time lag (Figures 2D and 2E). We found a high peak cross-correlation that increased as the peak dF/F increased and eventually saturated (Figures 2F and 2G). The peak of the cross-correlation decreased with distance from the looming center (Figures 2H and 2I). To resolve the spread of peak activation times across dendritic branches (cf. Figure S1B), we plotted the time lag at peak cross-correlation as a function of branch distance to the looming center (Figure 2J). The minimum lag was at the looming center, 0.11 ± 0.09 s. As expected from these results, the peak of the cross-correlation decreased with increasing peak time lag (Figure 2K). To summarize, the calcium signal at the looming center closely tracked the membrane potential with a delay of 110 ms; other branches experience nearly identical activation that is increasingly delayed with distance from the looming center.

### Muscarine Increases and Scopolamine Decreases Excitatory Dendritic Activation to Looming Stimuli

**Figure 3:**
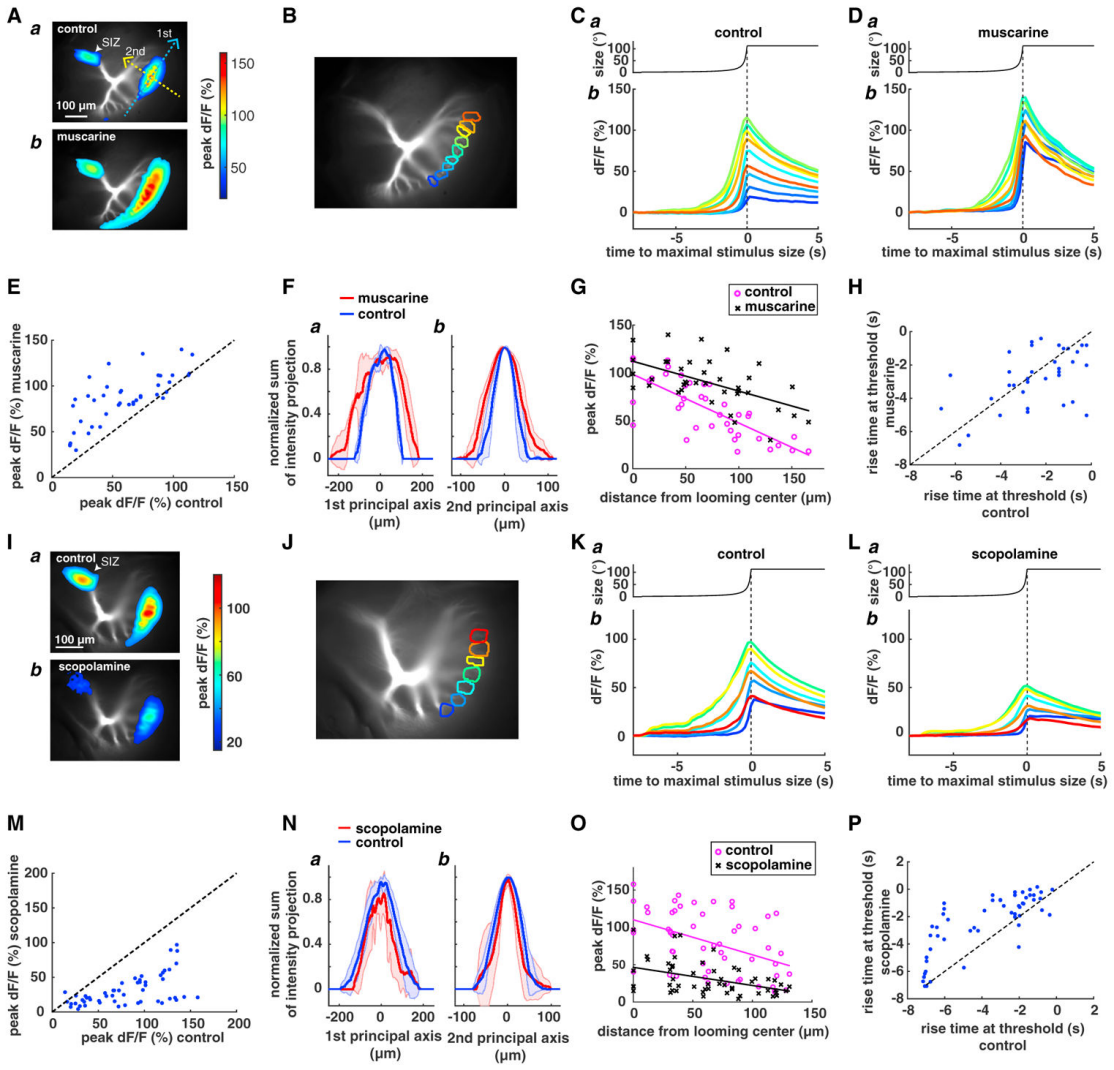
Effect of Muscarine and Scopolamine on Looming Responses (A) a: LGMD activation to looming, shown as peak dF/F at each activated pixel (color scale, right; average over 3 trials in 1 animal). Blue and yellow dashed arrows indicate the first and second principal axes. b: the same after puffing muscarine (average over 3 trials). (B) LGMD excitatory dendritic field with selected dendritic branches indicated by different colors. (C) a: stimulus angular size time course (*l/*|*v*| = 120 ms). b: average dF/F in response to this stimulus at each selected dendritic branch (B) in one animal. (D) The same as (C) after puffing muscarine. (E) Peak amplitude of dF/F on each dendritic branch after versus before muscarine (p = 4.84 × 10^−10^, paired t test). (F) a: projection of peak dF/F onto the first principal axis as a function of distance along the axis after normalizing to peak value without (blue) and with muscarine (red). Red and blue shadings, SD. FWHM before muscarine, 148.7 ± 13.3 μm (mean ± SD); after: 252.2 ± 13.2 μm (p = 0.0002, paired t test, before versus after). b: the same analysis but along the second principal axis. FWHM before muscarine: 61.2 ± 15.7 μm; after: 90.6 ± 18.2 μm (p = 0.0016). (G) Peak amplitude of dF/F on each dendritic branch as a function of branch distance to the looming center before (control) and after puffing muscarine (n = 5). Corresponding lines are linear regressions. Line slopes are significantly different (38 data points, p = 0.023, ANCOVA). (H) Rise time at threshold (as in Figure 1D) after versus before puffing muscarine (p = 0.31, paired t test). (I) a: LGMD activation to looming, shown as peak dF/F at each activated pixel (color scale, right; average over 3 trials in 1 animal). b: the same after puffing scopolamine (average over 3 trials). (J) LGMD excitatory dendritic field with selected dendritic branches indicated by different colors. (K) a: stimulus angular size time course (*l/*|*v*| = 120 ms). b: average dF/F at selected dendritic branches, with colors matched to (J). (L) The same as (K) after puffing scopolamine. In (K) and (L), the vertical dashed lines indicate the time of maximal stimulus size. (M) Peak dF/F amplitude on selected dendritic branches after versus before puffing scopolamine (p = 2.8 × 10^−15^, paired t test). (N) The same as (F) except that scopolamine was puffed. a: FWHM before scopolamine application: 168.3 ± 39.8 μm (mean ± SD); after: 141.2 ± 62.9 μm (p = 0.21, paired t test, before versus after). b, FWHM before scopolamine: 72.9 ± 13.1 μm; after: 60.8 ± 31.2 μm (p = 0.36). (O) The same as (G) except that scopolamine was puffed (53 data points, p = 0.22, ANCOVA). (P) Rise time at threshold (defined as in Figure 1D) after versus before puffing scopolamine (p = 1.92 × 10^−8^, paired t test). In (C) and (D), the vertical dashed line indicates the time of maximal stimulus size. In (E)–(H), n = 5. In (M)–(P), n = 7. See also Figure S3.

To study putative muscarinic acetylcholine receptors at the pre-synaptic terminals of the LGMD, we compared dendritic activation with looming before and after locally puffing muscarine in the lobula (Experimental Procedures). The activated dendritic area increased after puffing muscarine (Figure 3A). Calcium influx also increased close to the spike initiation zone (SIZ; Figure 3A), where voltage-gated calcium channels are activated by LGMD spiking (Peron and Gabbiani, 2009). Muscarine increased dF/F across dendritic branches (Figures 3B–3E). To quantify the increase in activated area, we computed its two principal axes relative to its center of mass and projected the dF/F along each of them (Figures S3A–S3E). The first and second principal axes approximately aligned with the ventral-dorsal and lateral-medial axes of the dendritic tree, allowing us to assess the change in dF/F relative to the axes of retinotopy and of looming stimulus expansion (elevation and azimuth in visual space; Zhu and Gabbiani, 2016). We computed the full width at half maximum (FWHM) of the activated area along the principal axes before and after drug application (Figure S3F). These curves broadened following muscarine application (Figure 3F). The peak dF/F decreased with increasing distance from the looming center before and after puffing muscarine (Figure 3G). The magnitude of the slope of the peak dF/F as a function of the distance from the looming center decreased after puffing muscarine. However, we did not find a difference between the rise time at threshold before and after puffing muscarine (Figure 3H). We also applied muscarine locally in the absence of visual stimuli and quantified the dF/F across dendritic segments (Figure S3G). Figures S3H and S3I depict such spontaneous dF/F traces before and after muscarine application. The increase in dF/F held across animals (Figure S3J).

We then compared activation on the excitatory dendritic field to looming stimuli before and after puffing scopolamine (Figures 3I–3P). Activation strongly decreased (Figure 3I), as did dF/F close to the SIZ (Figure 3I, arrowhead). On selected dendritic branches (Figure 3J), we found a uniform dF/F decrease (Figure 3K versus 3L). This result was consistent across animals (Figure 3M), although the change in the activation area was not significant after pooling across animals (Figure 3N, legend). The peak dF/F also decreased with increasing distance from the looming center after puffing scopolamine (Figure 3O). However, we did not observe a notable difference in the slope of the peak dF/F as a function of distance from the looming center before and after puffing scopolamine (Figure 3O). The rise time at threshold after puffing scopolamine was later than that before puffing (Figure 3P). Thus, the effects of scopolamine are qualitatively opposite to those of muscarine, in spite of quantitative differences.

Finally, we tested the effect of scopolamine on LGMD spiking by recording its activity to small transient stimuli flashed across the visual field (Figures S3K–S3M). Scopolamine decreased spiking by ~50%, suggesting that muscarinic acetylcholine receptors are tonically active because the flashes likely activated one or two ommatidia.

### Scopolamine and Muscarine Do Not Affect Presynaptic Dynamic Lateral Inhibition

**Figure 4:**
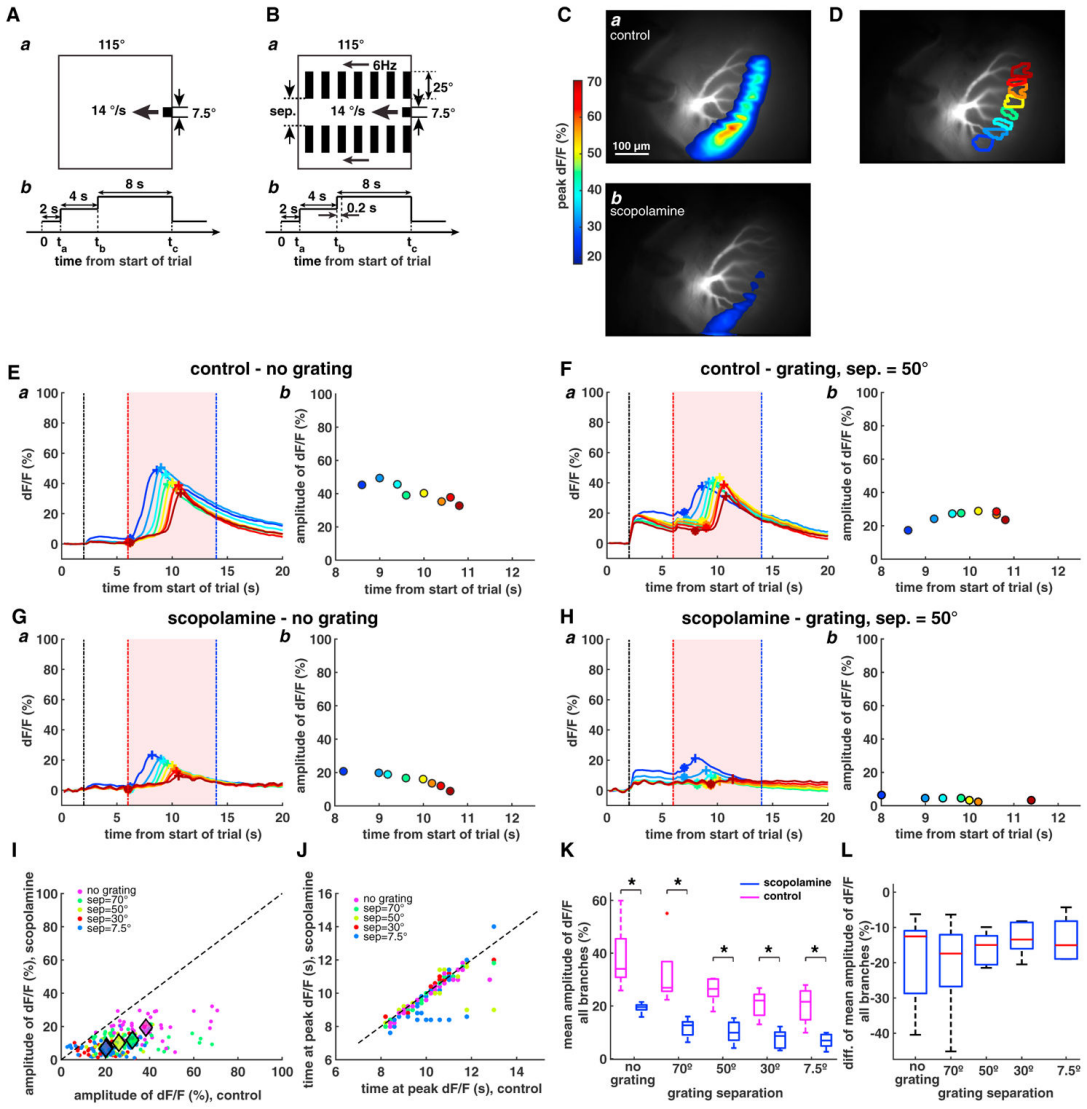
Test of the Influence of Scopolamine on Lateral Inhibition (A) a: stimulus schematics (control). b: stimulation time course. Time 0–t_a_, bright display; t_a_, stimulus on at right display edge; t_a_–t_b_, stimulus stays still; t_b_–t_c_, stimulus translates from right to left until it moves out of display. (B) a: stimulus schematics (lateral inhibition); flanking gratings temporal frequency, 6 luminance transitions per second, 90°/s. Grating width, 25°. sep., separation. b: stimulation time course. Time 0–t_a_, bright display; t_a_, stimulus on at right display edge, gratings on; t_a_–t_b_, stimulus stays still; t_b_–t_c_, gratings drift; t_b_+0.2 s–t_c_, stimulus translates from right to left until it moves out of display; t_c_, gratings stop drifting. (C) a: dendritic activation to translating stimulus. b: dendritic activation to the same stimulus after puffing scopolamine. (D) LGMD excitatory dendritic field with selected dendritic branches indicated by different colors. (E) a: average dF/F time course (over 3 trials) at each dendritic branch in (D) to the stimulus as in (A). Vertical dashed lines: black, time when stimulus appears; red, time when stimulus starts translating; blue, time when stimulus moves out of display. The asterisk and plus markers on each curve indicate calcium baseline and peak dF/F to the translating stimulus, respectively. b: dF/F amplitude computed by subtracting baseline (asterisks) from peak dF/F (plus symbols) for each curve in a (matched color) and plotted as a function of peak dF/F time. (F) a: average dF/F time course (over 3 trials) at each dendritic branch in (D) to the stimulus as in (B), with a 50° grating separation. Vertical dashed lines: black, time when stimulus and lateral gratings appear; red, time when lateral gratings start drifting (0.2 s before the small stimulus starts translating); blue, time when small visual stimulus moves outside of display and lateral gratings stop drifting. Asterisks and plus symbols as in (E). b: the same as (E), b. (G) The same as (E) after puffing scopolamine. (H) The same as (F) after puffing scopolamine. (I) dF/F amplitude on every selected dendritic branch after versus before puffing scopolamine (41 dendritic branches total, average of 3 trials per condition). Purple dots: translating stimuli, no drifting gratings. Green, light green, red, and blue dots: translating stimuli and lateral drifting gratings separated by 70°, 50°, 30°, and 7.5°, respectively. Purple, green, light green, red, and blue diamonds represent the mean (center of mass) of matched color dots, respectively. (J) Peak dF/F time on every selected dendritic branch after versus before puffing scopolamine with the same plotting conventions as in (I). (K) Boxplots of mean dF/F amplitudes for all branches in each animal to translating stimuli without and with drifting gratings. Blue and magenta, after and before puffing scopolamine, respectively (*p < 0.05, Wilcoxon signed-rank test, uncorrected for multiple comparisons; p = 0.025 for control and p = 0.0001 for scopolamine, one-way ANOVA; p = 0 for control versus scopolamine, two-way ANOVA). In this and subsequent boxplots, the central bar indicates the median, the top and bottom box edges indicate the 25^th^ and 75^th^ percentiles, the whiskers represent the extent of the data within 1.5 times the box height, and data points outside of this range (outliers) are plotted individually as crosses. (L) Boxplots of the difference of mean amplitudes for all branches in each animal after versus before puffing scopolamine to translating stimuli without and with drifting gratings. No significant statistical difference of means was found between any two groups (p = 0.66 one-way ANOVA). In (I)–(L), n = 5. See also Figure S4.

The LGMD’s response to a small translating object is inhibited by wide-field drifting stripes flanking it, provided they are sufficiently close (O’Shea and Rowell, 1975). This dynamic lateral (DL) inhibition protects the LGMD from habituation caused by background motion (e.g., during locomotion) and suppresses excitation early during looming (Gabbiani et al., 2002; Rind et al., 2016). The anatomical location of DL inhibition remains unknown. Because muscarinic acetylcholine receptors presynaptic to the LGMD are excitatory, they are unlikely to implement it. Because DL inhibition was predicted to be mediated by muscarinic acetylcholine receptors in presynaptic terminals to the LGMD (Rind and Simmons, 1998), we designed visual stimuli to probe this possibility. The baseline condition consisted of a small translating black square that appeared at one edge of the screen and later moved until completely outside of the display (Figure 4A; Video S2). This allowed us to separate the LGMD’s responses to the square’s appearance and its motion. Under the test conditions, the same stimulus moved, flanked by two gratings drifting in the same direction (Figure 4B). The distance between the two gratings was varied from 7.5° to 70°, yielding maximal and minimal activation of DL inhibition, respectively.

In response to the small translating stimulus, the dendritic area activated under control conditions was larger than that activated by looming stimuli (compare Figure 4Ca, with Figures 3A and 3I). This is surprising because looming stimuli elicit stronger LGMD firing than translating stimuli (Peron and Gabbiani, 2009). After puffing scopolamine, dendritic activation decreased (Figure 4Cb). On each selected dendritic branch (Figure 4D), dF/F in response to a translating stimulus was stronger than in response to the same stimulus flanked by drifting gratings (Figures 4E and 4F). Under test conditions, the amplitude of the dF/F decreased, but DL inhibition persisted (Figures 4G and 4H). We employed flanking gratings with 4 separations and found the peak dF/F to be smaller after than before puffing (Figure 4I; p < 10^−9^ for all stimuli, paired t test). The time at peak dF/F before and after puffing did not change for two separations and only slightly in the other 3 cases (median change: −1.1% at 70°, −4.2% at 7.5°, −1.8% for no grating; Figure 4J). The mean peak dF/F decreased with closer flanking gratings both before and after puffing (Figure 4K). However, the difference in mean peak dF/F showed no difference (Figure 4L, legend). This suggests that blocking muscarinic acetylcholine receptors presynaptic to the LGMD did not influence DL inhibition.

We tested the influence of muscarine on DL inhibition using the same protocol and found no effect (Figure S4). Thus, muscarinic acetylcholine receptors presynaptic to the LGMD do not appear to generate DL inhibition.

### Horizontal Band-Restricted Looming Stimuli Activate Lateral Branches Better Than Looming Stimuli

**Figure 5:**
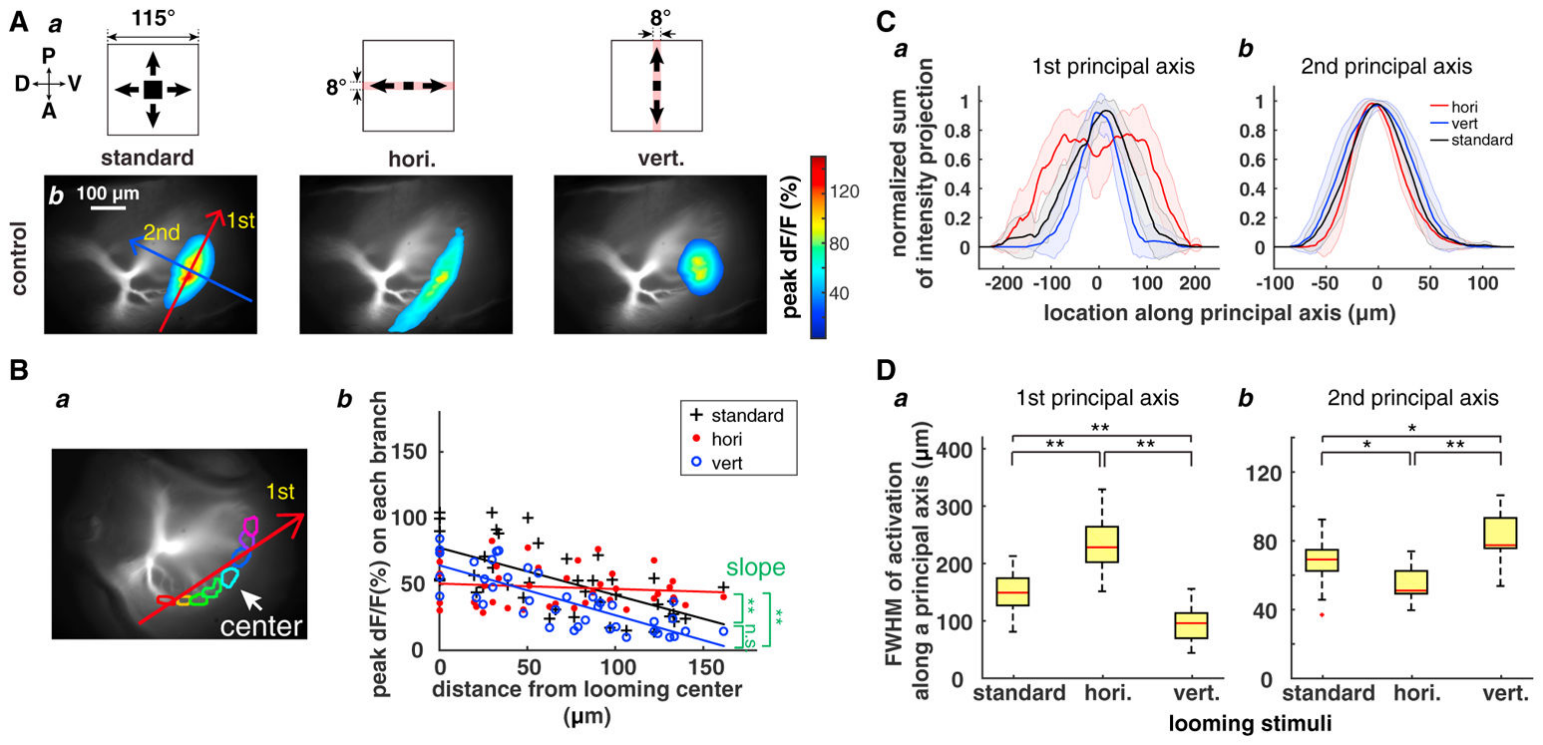
Calcium Responses to Looming Stimuli Restricted to Horizontal and Vertical Bands (A) a: from left to right, schematics of looming stimulus, HBR, and VBR looming stimuli, respectively. b: from left to right, LGMD dendritic field activation to the same stimuli. Red and blue arrows on left indicate the first and second principal axis, respectively. (B) a: 8 selected regions of interest (ROIs) represented by different colors in an example LGMD. The white arrow points to the looming center. The red arrow indicates the first principal axis of the looming response. b: peak dF/F in the 8 ROIs to SL (black crosses), HBR (red dots), and VBR (blue circles) stimuli. Solid lines indicate linear regressions (n = 5; **p < 0.01; n.s., not significant [i.e., p > 0.05], paired t test, ANCOVA test of slopes). (C) Projection of peak dF/F onto the first (a) and second (b) principal axes as a function of distance along the axis after normalizing to peak value in response to SL (black), HBR (red), and VBR (blue) stimuli, respectively. Red, black, and blue shadings, SD (n = 10). (D) Boxplots of the full width at half-maximum (FWHM) of the curves in (C) along the first (a) and second principal axis (b) to the SL or the HBR/VBR stimuli (n = 10, *p < 0.05, **p < 0.01, paired t test). Box plotting conventions are described in the legend to Figure 4K. See also Figures S5 and S6.

To study dendritic activation elicited by stimulus expansion along the cardinal axes, we designed looming stimuli restricted to horizontal (along the dorsal-ventral eye axis because of the 90° head rotation performed during the dissection; Experimental Procedures) or vertical (anterior-posterior axis) bands (Figure 5A). Because of the retinotopic mapping, the dF/F associated with horizontal band-restricted (HBR) stimuli mapped approximately onto the first principal axis of the response to a standard looming (SL) stimulus (Figure 5Ab, left). The vertical band-restricted (VBR) stimuli resulted in increased dF/F along the second principal axis. We found that the activation width of the HBR response was wider than that of the SL response along the first principal axis (Figure 5Ab), in spite of the higher peak dF/F for the latter stimulus. The activation width of the VBR response was narrower than that of the SL response, as expected. The peak dF/F decreased with distance from the looming center for the SL and VBR stimuli but not for the HBR one (Figure 5B). We compared dF/F along the first principal axis for the 3 stimuli and found that the HBR stimuli yielded the wider curve, followed by the SL and VBR stimuli (Figure 5Ca). Peak activation was not at the looming center for the HBR stimuli. Along the second principal axis, the width of activation was reversed, as expected, with the VBR stimuli yielding a slightly broader curve than SL and HBR stimuli (Figure 5Cb). The FWHM of the response to HBR stimuli was larger than that for the SL stimulus, whereas that of the VBR stimuli was smaller (Figure 5Db). The situation was reversed along the second principal axis. After applying muscarine, responses to all stimuli increased. Additionally, the differences between HBR and SL stimuli along the first principal axis were abolished, as were those of VBR and SL stimuli along the second principal axis (Figure S5). Motivated by the wide activation for HBR stimuli, we compared the activation elicited by small squares translating horizontally with that of looming stimuli. We again observed a broader activation for the translating squares along the first principal axis but not along the second one (Figure S6). The following set of experiments directly addresses the origin of the unexpectedly broad dendritic activation observed for spatially restricted stimuli.

### Presynaptic Muscarinic Acetylcholine Receptors Partially Counteract Global Inhibition Upstream of the LGMD

**Figure 6:**
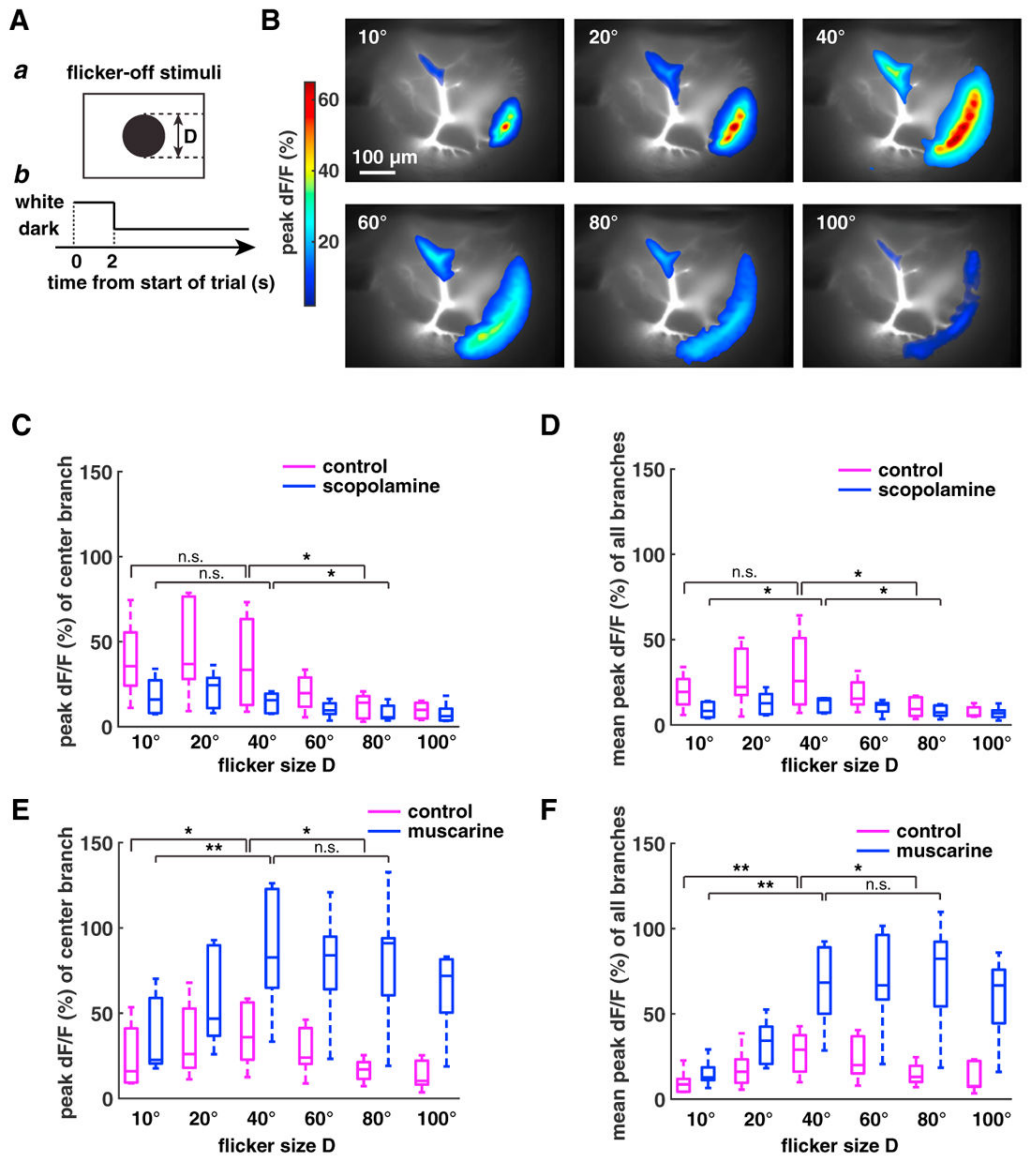
Effect of Scopolamine and Muscarine on Calcium Responses to Flicker-Off Stimuli (A) a: schematics of flicker-off stimuli with diameter D. b: time course of visual stimulus. Time 0–2 s, bright display; after 2 s, black stimulus flashed on at center of display. (B) LGMD activation to flicker-off stimuli of increasing size. (C) Boxplots of peak dF/F for the stimulus center branch to flicker-off stimuli of increasing size. Magenta and blue represent without and with scopolamine, respectively (n = 5; p = 0.0007 for control versus scopolamine, two-way ANOVA; p = 0.028 for control, p = 0.069 for scopolamine, one-way ANOVA). (D) Boxplots of mean peak dF/F over all branches to flicker-off stimuli with increasing size. Magenta and blue are as in (C) (n = 5; p = 0.0009 for control versus scopolamine, two-way ANOVA; p = 0.07 for control, p = 0.35 for scopolamine, one-way ANOVA). (E) Boxplots of peak dF/F for the stimulus center branch to flicker-off stimuli with increasing size. Magenta and blue represent without and with muscarine (n = 6; p = 0 for control versus muscarine, two-way ANOVA; p = 0.098 for control, p = 0.073 for muscarine, one-way ANOVA). Plotting conventions are as in (D). (F) Boxplots of mean peak dF/F over all branches to flicker-off stimuli with increasing size. Magenta and blue are as in (E) (n = 6; p = 0 for control versus muscarine, two-way ANOVA; p = 0.053 for control, p = 0.001 for muscarine, one-way ANOVA). In (C)–(F), *p < 0.05, **p < 0.01, n.s. not significant, paired t test. Box plotting conventions are described in the legend to Figure 4K.

To understand why the activation of lateral branches is weaker in response to SL than to HBR stimuli and to small translating squares, we designed flicker-off stimuli with different angular sizes (Figure 6A). The activated dendritic area initially increased with stimulus size (Figure 6B). However, for sizes >40°, the peak dF/F decreased as angular size further increased (Figure 6B). This suggests the existence of size-dependent inhibition upstream of the LGMD excitatory dendrites. We tested the influence of scopolamine and muscarine on this global inhibition. The peak dF/F of the center branch (Figure 6C) and the mean peak dF/F of all selected branches (Figure 6D) decreased as the size of the stimulus exceeded 40°. After puffing scopolamine, the mean peak dF/F was reduced for stimuli of a size <60° (Figure 6D). After puffing muscarine, the peak dF/F of the center branch (Figure 6E) and the mean peak dF/F of all branches (Figure 6F) increased as stimulus size increased from 10° to 40°, but it exhibited no decrease as stimulus size increased to 100°. This suggests that muscarinic acetylcholine receptors partially counteract global inhibition upstream of the LGMD.

### Blocking Muscarinic Lateral Excitation Decreases the LGMD’s Spatial Coherence Preference

**Figure 7:**
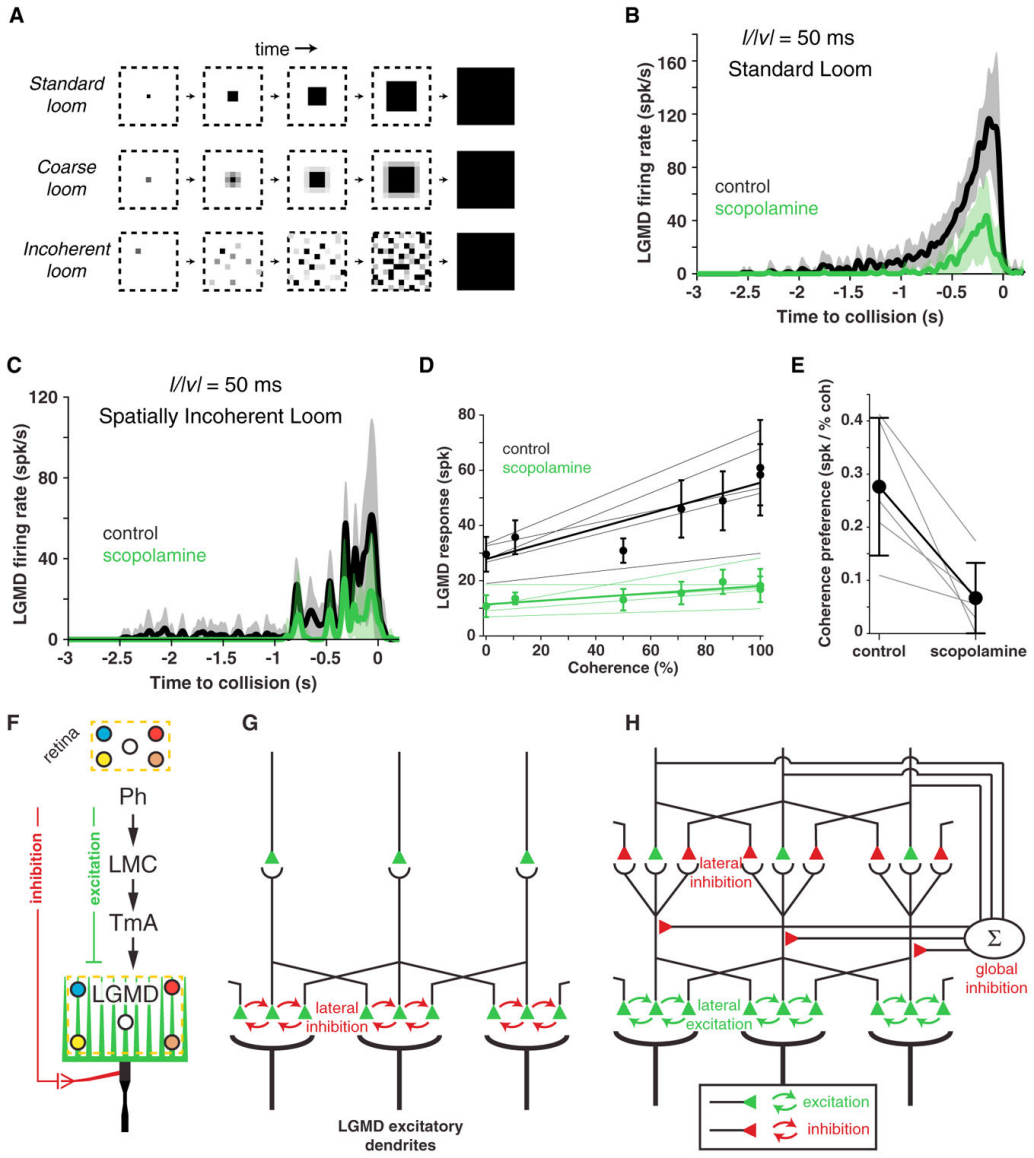
Scopolamine Reduced Coherence Preference and Schematic Models of Candidate Neural Circuits Presynaptic to Dendritic Field A of the LGMD (A) Illustration of coherent and incoherent stimuli. For coarse looms, grayscale levels are set so that the luminance in each coarse pixel is equal to that of standard looms in every frame. For reduced coherence looms, the spatial locations of coarse pixels were altered (Experimental Procedures). (B) LGMD’s response to looming stimuli (*l/*|*v*| = 50 ms) decreased after addition of scopolamine. The lines and shaded region indicate mean and ± 1 SD. (C) LGMD’s response to a spatially randomized stimulus also decreased after addition of scopolamine. Plotting conventions are as in (B). (D) LGMD responses increase with stimulus coherence (Pearson r = 0.90, p = 0.006), but this coherence preference decreased after scopolamine (p = 0.008, ANCOVA test of slopes). Thin lines indicate data from individual animals. Thick lines and points represent population average. Error bars indicate ± 1 mad. (E) Scopolamine caused a decrease in coherence preference (calculated from the slopes of the lines in C; p = 0.022, paired t test). There is an average decrease from 0.28 spikes to 0.07 spikes per percent coherence. Gray lines connect individual data points, and dots and whiskers indicate mean ± 1 SD. (F) Overview of the pathways impinging on the LGMD. Feedforward inhibition impinging on two other dendritic fields was not studied here. For feedforward excitation onto the excitatory dendritic field, colored dots illustrate retinotopy. Ph, photoreceptors; LMC, lamina large monopolar cells; TmA, transmedullary afferents. (G) Earlier model with lateral inhibition presynaptic to the LGMD. The LGMD dendrites are shown at the bottom, TmAs in the center, and axonal terminals of lamina neurons at the top. The synapses of axonal terminals of adjacent TmAs are connected through inhibitory synapses (red arrows). (H) Current model with lateral excitation presynaptic to the LGMD, normalizing inhibition, and upstream short-range lateral inhibition, with the same conventions as in (G). The exact locations of short-range lateral inhibition and global inhibition were not determined here, except that they are necessarily located upstream of the local lateral excitation. In (B)–(E), n = 5.

The LGMD is selective for the spatial coherence of approaching objects. This selectivity is partially explained by the interaction of hyperpolarization-activated, cyclic nucleotide-gated (HCN) channels with a slowly inactivating K^+^ current in the dendrites of the LGMD (Dewell and Gabbiani, 2018). Because presynaptic lateral excitation is likely local (Rind et al., 2016; Zhu and Gabbiani, 2016), we reasoned that it might increase responses to coherently expanding objects. To test this, we designed stimuli with varying degrees of coherence by dividing the screen in an array of 2° wide “coarse pixels” roughly spanning the receptive field of a single ommatidium. We replaced the dark edge motion associated with a looming stimulus expanding across each coarse pixel by an equivalent uniform decrease in luminance. The resulting stimulus, which we call a “coarse loom” (Figure 7A), elicits LGMD responses nearly identical to those of SL stimuli (Jones and Gabbiani, 2010; Dewell and Gabbiani, 2018). Stimuli with decreasing coherence can be generated from coarse looms by randomly displacing individual coarse pixels increasingly far from their original position (Figure 7A). Retinotopy implies that, as stimulus coherence increases, synaptic activation in the LGMD dendrites becomes more local.

We presented such stimuli while recording the firing rate of the LGMD before and after blocking it with scopolamine. The timing of LGMD firing remained similar after adding scopolamine, but with much lower firing rates (Figures 7B and 7C). Responses to SL stimuli decreased from 58 ± 16 to 17 ± 7 spikes after addition of scopolamine (mean ± SD, p = 0.004, paired t test). Responses to incoherent stimuli were reduced as well (Figure 7C), from 30 ± 8 to 11 ± 6 spikes (p = 0.017). For every animal, there was a decrease in LGMD firing (p ≤ 0.004, analysis of covariance [ANCOVA]) and a reduced coherence-dependent increase in firing (Figure 7D). Coherence preference decreased from 0.28 to 0.07 spikes per percent coherence (Figure 7E). Because scopolamine reduced responses at all coherence levels, we calculated the coherence-specific reduction by subtracting from the reduction in coherent looming response the reduction in incoherent response and by expressing it as a percentage of the control looming response. We found a 37.1% ± 17.8% (mean ± SD) specific reduction for responses to coherent looming stimuli (p = 0.01, paired t test). These results indicate that lateral excitation shapes the LGMD’s preference for coherently expanding stimuli. Additionally, they suggest that lateral excitation is implemented locally because the effects of scopolamine increase with coherence and, thus, as synaptic activation of the LGMD dendrites becomes increasingly local.

In summary, our results and earlier anatomical ones (Rind et al., 2016) suggest that feedforward excitation impinging on the LGMD (Figure 7F) is differently organized than what was assumed up to now (Figure 7G). Figure 7H presents the simplest model consistent with these data. In this model, lateral excitation is local and presynaptic to the LGMD, whereas lateral inhibition (Figure 4) is upstream, as is global inhibition (Figure 6).

## DISCUSSION

Little is known about how networks converging onto collision-detecting neurons are wired to shape their selectivity. Using calcium imaging, we investigated how connectivity among presynaptic excitatory inputs to the LGMD shapes its responses. We provide evidence for two types of connectivity patterns: lateral excitation and global (normalizing) inhibition. Lateral excitation promotes responses to coherently expanding stimuli, tuning the neuron to its preferred features. Global inhibition likely keeps excitation within the dynamic range of the neuron. Together with DL inhibition suppressing background motion noise (O’Shea and Rowell, 1975), these experiments show that the presynaptic interactions among excitatory inputs to the LGMD are governed by a complex, but interpretable, set of rules.

Calcium imaging was effective at establishing the excitatory effect of muscarinic acetylcholine receptors on synaptic excitation to the LGMD. These excitatory interactions could not be evidenced in earlier experiments where only two adjacent ommatidia were stimulated (Jones and Gabbiani, 2010; Zhu and Gabbiani, 2016). Earlier experiments showed that blocking nicotinic acetylcholine receptors abolished calcium responses in LGMD dendrites upon subsequent iontophoresis of acetylcholine (Peron et al., 2009; Figure S1). This suggests that LGMD dendrites do not possess excitatory muscarinic acetylcholine receptors because their effects usually rely on calcium signaling.

In mammals, 5 types of muscarinic acetylcholine receptors (m1–m5) have been found, of which m1, m3, and m5 are coupled to G protein members of the G_q/11_ family, mostly resulting in excitatory effects. Two other muscarinic acetylcholine receptors types (m2 and m4) couple to G proteins of the G_i/0_ family, which leads to presynaptic or postsynaptic inhibition (Caulfield, 1993; Brown, 2010). In flies and other arthropods, 3 types of muscarinic acetylcholine receptors have been found: types A, B, and C (Xia et al., 2016). Type A resembles most of the vertebrate muscarinic acetylcholine receptors and is activated by acetylcholine and muscarine and blocked by atropine and scopolamine. Type B is also activated by acetylcholine but insensitive to muscarine and not blocked by classical antagonists (Collin et al., 2013). The type A muscarinic acetylcholine receptor is coupled to G_q/11_ type proteins, whereas type B is coupled to G_i/0_ (Ren et al., 2015). Type C resembles type A (Xia et al., 2016). Our results suggest that the muscarinic acetylcholine receptors presynaptic to the LGMD are either of type A or C. Their excitatory effects might result from inhibition of presynaptic M channels or other potassium channels (Brown, 2010). In *Drosophila*’s visual system, T4 and T5 cells, directionally selective neurons that synapse onto lobula plate tangential cells, both express transcripts of nicotinic and muscarinic acetylcholine receptors (Shinomiya et al., 2014). Although their role remains unknown, our results suggest that muscarinic acetylcholine receptor activation likely last >100 ms, a long time relative to that relevant for directional motion detection (Behnia et al., 2014). However, muscarinic acetylcholine receptors may contribute to other visual detection tasks requiring local modulation of activity on a slow timescale.

The most likely localization of muscarinic acetylcholine receptors is between adjacent presynaptic terminals of the LGMD because these synapses are cholinergic, and none have been reported elsewhere (Rind et al., 2016). Because of the retinotopic projection of these axons onto LGMD dendrites, their effects would have to be local (Figure 7H). This is consistent with the coherence-dependent effect of scopolamine (Figures 7A–7E). Our results do not rule out that some lateral presynaptic connections between LGMD excitatory afferents could be inhibitory. If this were the case, these inhibitory synapses would be considerably weaker than the excitatory ones because we could not detect any decrease in postsynaptic calcium upon muscarine application. Such inhibitory synapses could not be GABAergic (Rind and Simmons, 1998) and could not implement lateral inhibition if cholinergic (Figure 4).

Our experiments yielded several unexpected results. First, calcium activation outlasted the time interval over which a looming stimulus swept across the retinotopic region associated with a given dendritic branch. At the dendritic looming center, relative fluorescence changes outlasted the stimulus by as much as 1 s (Figures 1B and 1C). Two observations help explain this. First, the continued rise of dF/F immediately after the stimulus has left the red-shaded region in Figures 1B and 1C is caused in part by overlapped dendritic activation (red double arrow in Figure 1C; Zhu and Gabbiani, 2016). Second, although the horizontal edges of the stimulus leave the retinotopic region associated with the dendritic looming center, its vertical edges continue to stimulate the same dendritic branch and, thus, cause additional calcium entry (Figures 5A and 5C). Although muscarine and scopolamine consistently affected the strength of calcium signals (Figure 3), their effect on rise time differed (Figures 3H and 3P). It thus remains unclear whether muscarinic excitatory lateral interactions also contribute to responses beyond the region of dendritic overlap.

A second unexpected result was the excitatory nature of muscarinic acetylcholine receptor-mediated lateral interactions presynaptic to the LGMD. Up to now, the only demonstrated lateral interactions presynaptic to the LGMD were those of DL inhibition (O’Shea and Rowell, 1975). It was thus plausible that they would be implemented by the lateral connections evidenced at the level of the LGMD (Rind and Simmons, 1998). Our results (Figures 4 and S4) indicate that this is not the case and that DL inhibition is most likely located further upstream.

A third unexpected result is the presence of size-dependent, global inhibition upstream of lateral excitation. Global inhibition explains why the dendritic activation width for an SL stimulus is smaller than that observed for an HBR stimulus or for a small translating stimulus. The function of global inhibition is likely to normalize the strength of the excitatory inputs impinging on the LGMD. As a looming stimulus expands, the number of activated ommatidia grows nearly exponentially, and the increase in edge velocity increases the strength of individual synaptic inputs as well (Jones and Gabbiani, 2010). Without normalization, excitation would grow more than 10^3^ fold. Global inhibition would normalize these inputs into the dynamic range of the LGMD. The results reported in Figure 1F are consistent with this hypothesis. A similar role for normalizing global inhibition has been documented in other sensory systems (Carandini and Heeger, 2011).

It is likely that several of our stimuli, particularly the larger ones, activated feedforward inhibition onto dendritic field C of the LGMD. Feedforward inhibition would hyperpolarize the membrane potential in dendritic field A and increase the calcium driving force and influx through nicotinic acetylcholine receptors. Consequently, our experiments likely underestimated the strength of global inhibition for such stimuli. Feedforward inhibition curtails the LGMD firing rate as the size of looming stimuli increases beyond an angular threshold (unpublished data). Our results suggest that global inhibition fulfills a different role (Wang et al., 2018).

Because lateral excitation is most likely local, requires activation of several neighboring ommatidia, and occurs on a relatively slow timescale, it might help discriminate between coherently and incoherently expanding looming stimuli. We confirmed this by showing that coherence sensitivity was diminished following blocking of lateral excitation (Figure 7). Local lateral excitation thus helps tune the LGMD to looming stimuli in addition to conductances located within the LGMD’s dendrites (Dewell and Gabbiani, 2018). However, presynaptic mechanisms play a somewhat smaller role (median coherence-specific reduction of 57% after blocking HCN channels, computed from Dewell and Gabbiani, 2018, versus 34% median specific reduction after scopolamine addition reported here).

These results have implications for other collision detection circuits. Particularly interesting will be to determine how they discriminate between coherent and incoherent looming stimuli and whether the biophysical mechanisms involved resemble those implemented in the locust visual system.

## EXPERIMENTAL PROCEDURES

### Preparation

Experiments were carried out on locusts 3–4 weeks past the final molt. Animals were mounted dorsal side up on a custom holder with their heads rotated 90° to make the anterior side point downward. The head and neck were bathed in locust saline, except for the right eye, which was used for visual stimulation. The head capsule was opened dorso-frontally between the two eyes. The gut and the muscles in the head capsule were removed to reduce brain movement. The head was detached from the body, leaving only the two nerve cords and 4 tracheas attached. The right optic lobe was desheathed with fine forceps. A metal holder was placed under the brain and the right optic lobe to elevate and stabilize them.

### Visual Stimulation

Visual stimuli were generated with the Psychtoolbox (PTB-3) and MATLAB (MathWorks, Natick, MA). A digital light processing projector (DLP LightCrafter 4500, Texas Instruments, Dallas, TX) and a diffuser screen (non-adhesive stencil film, 0.1 mm thick) were used to provide the visual stimuli. The projector was programmed in pattern sequence mode with bitmap images in green color, which achieved a refresh rate of 243 Hz. The brightness of the display before presenting looming stimuli was 38.7 cd/m^2^. The screen brightness at the level used for off stimuli was 0.06 cd/m^2^. The brightness of the screen was calibrated and linearized by loading a normalized gamma table to the Psychtoolbox. The screen was placed 25 mm away from the locust eye and measured 79 × 79 mm. For the experiments presented in Figure 7, a cathode ray tube monitor refreshed at 200 frames/s was used for stimulus display. The monitor was calibrated to ensure linear, 6-bit resolution over luminance levels. A 100-to 120-s delay was used between stimuli, and locusts were repeatedly brushed and exposed to light flashes and high-frequency sounds to decrease habituation. Coarse looming stimuli were generated as in Dewell and Gabbiani (2018). Briefly, the stimulation monitor was first pixelated with a spatial resolution approximating that of the locust eye (2 × 2°), referred to as coarse pixels. Each coarse pixel’s luminance followed the same time course as that elicited by the edge of the simulated approaching object sweeping over its area. To alter spatial coherence, a random two-dimensional Gaussian jitter with zero mean was added to each coarse pixel screen location. The jittered positions were rounded to the nearest available coarse pixel location on the screen to prevent coarse pixels from overlapping. The SD of the Gaussian was altered between 0° and 80° to control the amount of shifting and the resulting spatial coherence of the randomized stimulus.

### Staining with Calcium Indicators

At the beginning of an experiment, the LGMD was impaled with a sharp intracellular electrode (230–300 MΩ) containing 3 μl of 2 M potassium chloride and 1 μL of 5 mM OGB-1 or OGB-5N (hexapotassium salt; Thermo Fisher Scientific, Waltham, MA). Because of the stronger signal amplitude of OGB-1 compared with OGB-5N, OGB-1 was used in all experiments that investigated the effects of muscarine or scopolamine. For other experiments, we used OGB-5N because its kinetics were faster than those of OGB-1 (Figure S2). The kinetics of both dyes have been characterized in controlled environments, but they are likely affected by parameters beyond our control *in vivo* (e.g., intracellular pH). Iontophoresis of OGB-1 or OGB-5N was achieved with current pulses of −3 nA, alternating between 1 s on and 1 s off, that lasted 6 min. The pulses were delivered by an Axoclamp 2B amplifier (Molecular Devices, Sunnyvale, CA). The LGMD was identified through its 1:1 spike correspondence with that of the descending contralateral movement detector (DCMD) (O’Shea and Williams, 1974), recorded extracellularly from the nerve cord with hook electrodes.

### Imaging, Electrophysiology, Pharmacology, and Data Acquisition

We employed wide-field calcium imaging during visual stimulation experiments. The calcium indicators were excited by a 100-W mercury short arc lamp, and the resulting fluorescence was measured by a charge-coupled device (CCD) camera at a frame rate of 5 Hz (Rolera XR, Qimaging, Surrey, BC, Canada). We used a 16×/0.8 numerical aperture (NA) water immersion objective lens for imaging (CFI75 LWD 16XW, Nikon Instruments). 3 to 5 trials were averaged for each stimulus.

Scopolamine and muscarine were prepared at 6 mM in locust saline containing fast green (0.5%) to visually monitor the affected region. They were puffed using a pneumatic picopump (PV830, WPI, Sarasota, FL). Injection pipettes had tip diameters of ~2 μm and were visually positioned between the excitatory dendritic field of the LGMD and its most superficial inhibitory field with a micromanipulator (fields A and C; O’Shea and Williams, 1974). Brief air pulses with a pressure of ~6 psi caused drug ejection into the optic lobe. Spread of fast green was monitored to ensure that drugs diffused through the excitatory dendritic field. The visual stimuli were presented with an inter-stimulus interval of 5–6 min. Muscarine was puffed immediately before each stimulus, provided 5 s after pressure ejection ended. Based on previous calculations of drug dilution within the optic lobe (see Materials and Methods in Dewell and Gabbiani, 2018), our best estimate for the final drug concentration at the LGMD is ~100 μM.

Intracellular LGMD and extracellular DCMD recordings were performed simultaneously with calcium imaging in some animals. After identification of the location in the LGMD’s excitatory dendritic branches that had been stained with the calcium indicator OGB-5N or OGB-1, a sharp electrode (25 MOmega;) containing 3 μL of 2 M potassium acetate, 0.5 M potassium chloride, and 0.1 mM Alexa 488 was inserted into the excitatory dendrites under a 16× objective lens for recording. Intracellular signals were amplified in bridge mode with an Axoclamp 2B and an instrumental amplifier (Brownlee Precision 440; NeuroPhase, Palo Alto, CA). LGMD and DCMD signals were acquired with a sampling rate of 5 kHz by a data acquisition card (PCI 6110; National Instruments, Austin, TX).

### Data Analysis

Data analysis was performed with MATLAB. The relative fluorescence change was calculated as dF/F(t) = (F(t)−F_0_)/F_0_, with the baseline fluorescence F_0_ being the average of the first 10 frames (2 s) before the visual stimulus. For peak dF/F spatial maps (e.g., Figure 3A), the value of dF/F(t) at each pixel was calculated as the median over a 5 × 5 pixel area centered at the given pixel (0.9 μm/pixel). After median filtering, only suprathreshold pixels were used, defined as having a peak dF/F > 180% of the maximum noise of dF/F at that pixel. The maximum (baseline) noise at each pixel was computed as the maximum of the dF/F values within 2 s before the onset of the visual stimulus. The threshold value of 180% was selected to eliminate pixels resulting in falsely positive dF/F outside the dendritic branches based on earlier 2-photon imaging experiments with high spatial resolution (Zhu and Gabbiani, 2016). The peak dF/F at the pixels above threshold were retained and passed through a Gaussian filter with an SD of 5 pixels. In Figure 1D, we selected the threshold value (dashed line) to be well above the baseline noise level so that calcium signals were unambiguously increasing at those time points. The results in Figures 1E and 1F are robust to changes of the threshold.

To map the dendritic receptive fields of square flashes at 5 locations on the screen, we measured the peak dF/F in response to individual flashes. For this purpose, we filtered the pixels that were above the noise level, then computed the maximum of the peak dF/F over all the pixels, and retained pixels above 4% of the maximum. This additional thresholding step was necessary because of the small amplitude of peak dF/F in response to square flashes. The threshold was selected to eliminate falsely positive dF/F pixels outside of the dendritic branches. The branches that were covered by the retained pixels were taken to be the dendritic receptive fields of that square flash.

The principal axes of the peak dF/F spatial map were computed by scaling and rounding the intensity of the peak dF/F at each pixel into integers. We took these integers for each pixel as the number of identical data points with coordinates matching those of that pixel. We performed a principal-component analysis on these data points to extract the first and the second principal components. We rotated the peak dF/F spatial map to make the first or second principal axis horizontal and summed the values of peak dF/F along the vertical axis to get the projected values along each principal axis.

To make plots, the membrane potential was first median-filtered over a time window of 30 ms and down-sampled 100 times (to 50 Hz).

### Statistics

For paired comparison of the control and scopolamine groups, we performed a nonparametric one-tailed Wilcoxon signed-rank test. For responses to small flashes (Figures S3K–S3M), a non-parametric Wilcoxon rank-sum test was conducted on the number of spikes in response to individual stimuli before and after addition of scopolamine. To compare statistical differences between the means of groups stimulated with gratings of varying separations, we performed a one-way ANOVA. For comparing the slopes of two regression lines, we performed an ANCOVA. A two-way ANOVA was performed to compare statistical differences between the control and the scopolamine/muscarine groups, each of which had different levels (types of visual stimuli). For comparisons using paired t test, normality was tested with a Lilliefors test with an alpha level of 0.2. For responses to stimuli with varying coherence (Figures 7A–7E), all changes were normally distributed, and a paired t test was used. We use the abbreviation mad for maximal absolute deviation.

## Supplementary Material

1

2

3

## Highlights

- Calcium fluorescence closely tracks dendritic membrane potential for looming stimuli
- Local muscarinic receptors between presynaptic LGMD afferents mediate excitation
- Muscarinic receptors help distinguish coherent from incoherent expanding objects
- Global inhibition normalizes the excitatory synaptic input to the LGMD during looming

## References

[R1] Behnia R, Clark DA, Carter AG, Clandinin TR, Desplan C (2014). Processing properties of ON and OFF pathways for Drosophila motion detection. Nature.

[R2] Brown DA (2010). Muscarinic acetylcholine receptors (mAChRs) in the nervous system: some functions and mechanisms. J Mol Neurosci.

[R3] Carandini M, Heeger DJ (2011). Normalization as a canonical neural computation. Nat Rev Neurosci.

[R4] Caulfield MP (1993). Muscarinic receptors–characterization, coupling and function. Pharmacol Ther.

[R5] Collin C, Hauser F, Gonzalez de Valdivia E, Li S, Reisenberger J, Carlsen EM, Khan Z, Hansen NO, Puhm F, Søndergaard L (2013). Two types of muscarinic acetylcholine receptors in Drosophila and other arthropods. Cell Mol Life Sci.

[R6] de Vries SEJ, Clandinin TR (2012). Loom-sensitive neurons link computation to action in the Drosophila visual system. Curr Biol.

[R7] Dewell RB, Gabbiani F (2018). Biophysics of object segmentation in a collision-detecting neuron. eLife.

[R8] Dunn TW, Gebhardt C, Naumann EA, Riegler C, Ahrens MB, Engert F, Del Bene F (2016). Neural Circuits Underlying Visually Evoked Escapes in Larval Zebrafish. Neuron.

[R9] Fotowat H, Gabbiani F (2007). Relationship between the phases of sensory and motor activity during a looming-evoked multistage escape behavior. J Neurosci.

[R10] Fotowat H, Gabbiani F (2011). Collision detection as a model for sensory-motor integration. Annu Rev Neurosci.

[R11] Gabbiani F, Krapp HG, Laurent G (1999). Computation of object approach by a wide-field, motion-sensitive neuron. J Neurosci.

[R12] Gabbiani F, Mo C, Laurent G (2001). Invariance of angular threshold computation in a wide-field looming-sensitive neuron. J Neurosci.

[R13] Gabbiani F, Krapp HG, Koch C, Laurent G (2002). Multiplicative computation in a visual neuron sensitive to looming. Nature.

[R14] Gabbiani F, Cohen I, Laurent G (2005). Time-dependent activation of feed-forward inhibition in a looming-sensitive neuron. J Neurophysiol.

[R15] Gray JR, Lee JK, Robertson RM (2001). Activity of descending contralateral movement detector neurons and collision avoidance behaviour in response to head-on visual stimuli in locusts. J Comp Physiol A Neuroethol Sens Neural Behav Physiol.

[R16] Hatsopoulos N, Gabbiani F, Laurent G (1995). Elementary computation of object approach by wide-field visual neuron. Science.

[R17] Jones PW, Gabbiani F (2010). Synchronized neural input shapes stimulus selectivity in a collision-detecting neuron. Curr Biol.

[R18] Judge S, Rind F (1997). The locust DCMD, a movement-detecting neurone tightly tuned to collision trajectories. J Exp Biol.

[R19] Liu YJ, Wang Q, Li B (2011). Neuronal responses to looming objects in the superior colliculus of the cat. Brain Behav Evol.

[R20] O’Shea M, Rowell CH (1975). Protection from habituation by lateral inhibition. Nature.

[R21] O’Shea M, Williams JL (1974). The anatomy and output connection of a locust visual interneurone: the lobular giant movement detector (LGMD) neurone. J Comp Physiol.

[R22] Oliva D, Tomsic D (2014). Computation of object approach by a system of visual motion-sensitive neurons in the crab Neohelice. J Neurophysiol.

[R23] Peron S, Gabbiani F (2009). Spike frequency adaptation mediates looming stimulus selectivity in a collision-detecting neuron. Nat Neurosci.

[R24] Peron SP, Jones PW, Gabbiani F (2009). Precise subcellular input retinotopy and its computational consequences in an identified visual inter-neuron. Neuron.

[R25] Preuss T, Osei-Bonsu PE, Weiss SA, Wang C, Faber DS (2006). Neural representation of object approach in a decision-making motor circuit. J Neurosci.

[R26] Ren GR, Folke J, Hauser F, Li S, Grimmelikhuijzen CJ (2015). The A- and B-type muscarinic acetylcholine receptors from *Drosophila melanogaster* couple to different second messenger pathways. Biochem Biophys Res Commun.

[R27] Rind FC, Leitinger G (2000). Immunocytochemical evidence that collision sensing neurons in the locust visual system contain acetylcholine. J Comp Neurol.

[R28] Rind FC, Simmons PJ (1992). Orthopteran DCMD neuron: a reevaluation of responses to moving objects. I Selective responses to approaching objects. J Neurophysiol.

[R29] Rind FC, Simmons PJ (1998). Local circuit for the computation of object approach by an identified visual neuron in the locust. J Comp Neurol.

[R30] Rind FC, Wernitznig S, Pölt P, Zankel A, Gütl D, Sztarker J, Leitinger G (2016). Two identified looming detectors in the locust: ubiquitous lateral connections among their inputs contribute to selective responses to looming objects. Sci Rep.

[R31] Santer RD, Rind FC, Stafford R, Simmons PJ (2006). Role of an identified looming-sensitive neuron in triggering a flying locust’s escape. J Neurophysiol.

[R32] Shang C, Liu Z, Chen Z, Shi Y, Wang Q, Liu S, Li D, Cao P (2015). BRAIN CIRCUITS. A parvalbumin-positive excitatory pathway to trigger fear responses in mice. Science.

[R33] Shinomiya K, Karuppudurai T, Lin TY, Lu Z, Lee CH, Meinertzhagen IA (2014). Candidate neural substrates for off-edge motion detection in Drosophila. Curr Biol.

[R34] Sun H, Frost BJ (1998). Computation of different optical variables of looming objects in pigeon nucleus rotundus neurons. Nat Neurosci.

[R35] Temizer I, Donovan JC, Baier H, Semmelhack JL (2015). A visual pathway for looming-evoked escape in larval zebrafish. Curr Biol.

[R36] von Reyn CR, Breads P, Peek MY, Zheng GZ, Williamson WR, Yee AL, Leonardo A, Card GM (2014). A spike-timing mechanism for action selection. Nat Neurosci.

[R37] von Reyn CR, Nern A, Williamson WR, Breads P, Wu M, Namiki S, Card GM (2017). Feature Integration Drives Probabilistic Behavior in the Drosophila Escape Response. Neuron.

[R38] Wang H, Dewell RB, Zhu Y, Gabbiani F (2018). Neural code subserving feed-forward inhibition in a collision detection circuit. Curr Biol.

[R39] Wilson M (1975). Angular sensitivity of light and dark adapted locust retinula cells. J Comp Physiol.

[R40] Wu M, Nern A, Williamson WR, Morimoto MM, Reiser MB, Card GM, Rubin GM (2016). Visual projection neurons in the *Drosophila* lobula link feature detection to distinct behavioral programs. eLife.

[R41] Xia RY, Li MQ, Wu YS, Qi YX, Ye GY, Huang J (2016). A new family of insect muscarinic acetylcholine receptors. Insect Mol Biol.

[R42] Zhao X, Liu M, Cang J (2014). Visual cortex modulates the magnitude but not the selectivity of looming-evoked responses in the superior colliculus of awake mice. Neuron.

[R43] Zhu Y, Gabbiani F (2016). Fine and distributed subcellular retinotopy of excitatory inputs to the dendritic tree of a collision-detecting neuron. J Neurophysiol.

