# Supplementary material for: Pre-synaptic Muscarinic Excitation Enhances the Discrimination of Looming Stimuli in a Collision-Detection Neuron
Source: Cell Rep. Author manuscript; Available in PMC 2018 Jun 12. (PMC5997271; doi:10.1016/j.celrep.2018.04.079)
Supplement: 1 [file NIHMS972536-supplement-1.pdf]

Cell Reports, Volume *23*

Supplemental Information

Pre-synaptic Muscarinic Excitation Enhances the Discrimination of Looming Stimuli in a Collision-Detection Neuron

Ying Zhu, Richard B. Dewell, Hongxia Wang, and Fabrizio Gabbiani

Figure S1

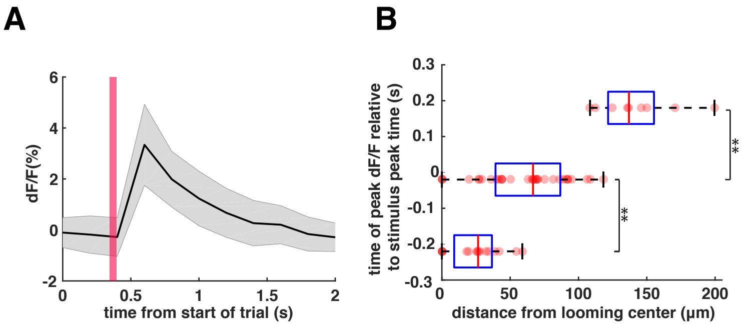
A
B
dF/F(%)
time from start of trial (s)
time of peak dF/F relative to stimulus peak time (s)
distance from looming center (μm)
**
**

Figure S2

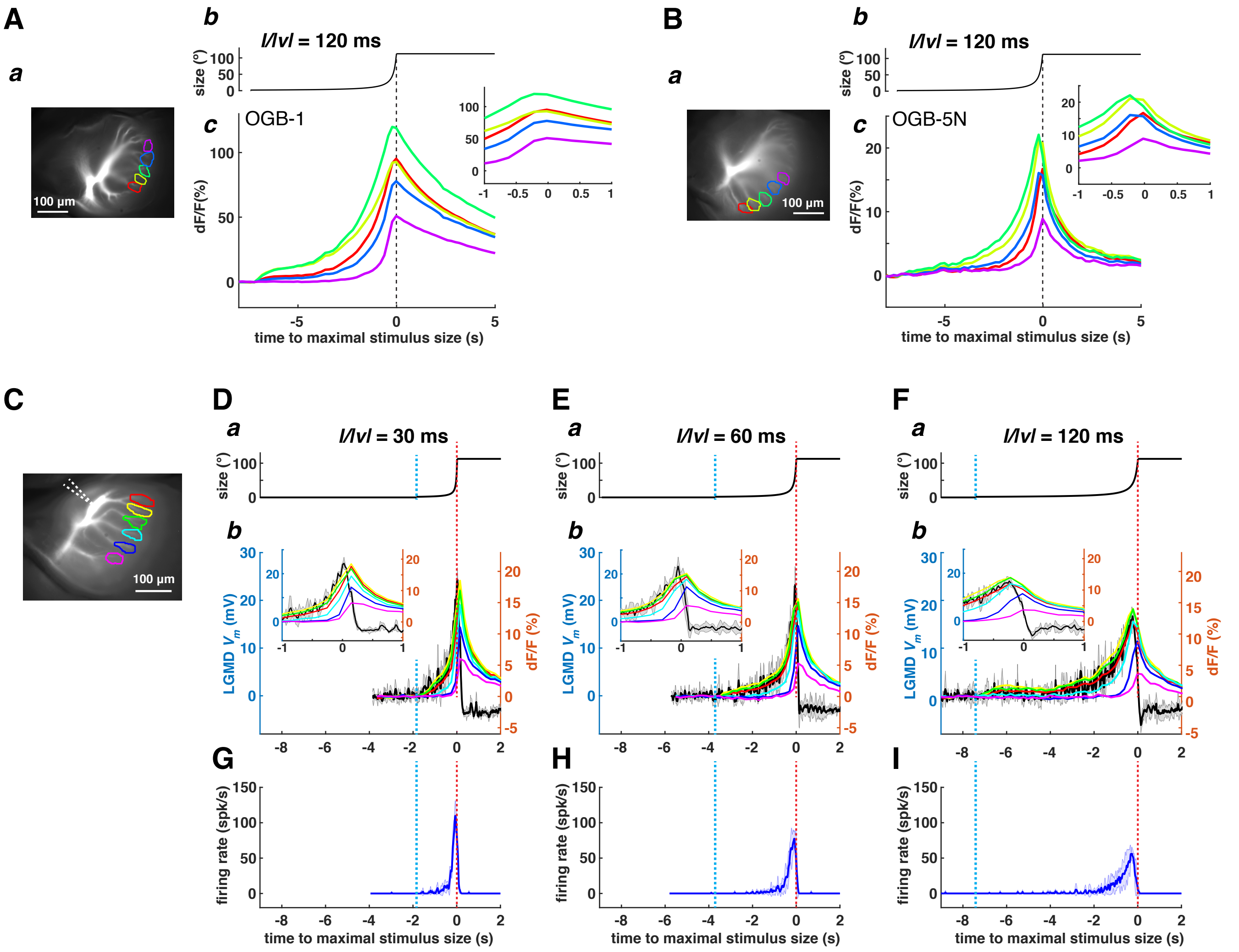

A
a
b
l/|v| = 120 ms
size (°)
c
OGB-1
dF/F(%)
time to maximal stimulus size (s)
100 µm
B
OGB-5N
C
D
E
F
l/|v| = 30 ms
l/|v| = 60 ms
LGMD V_m (mV)
dF/F (%)
G
H
I
firing rate (spk/s)

Figure S3

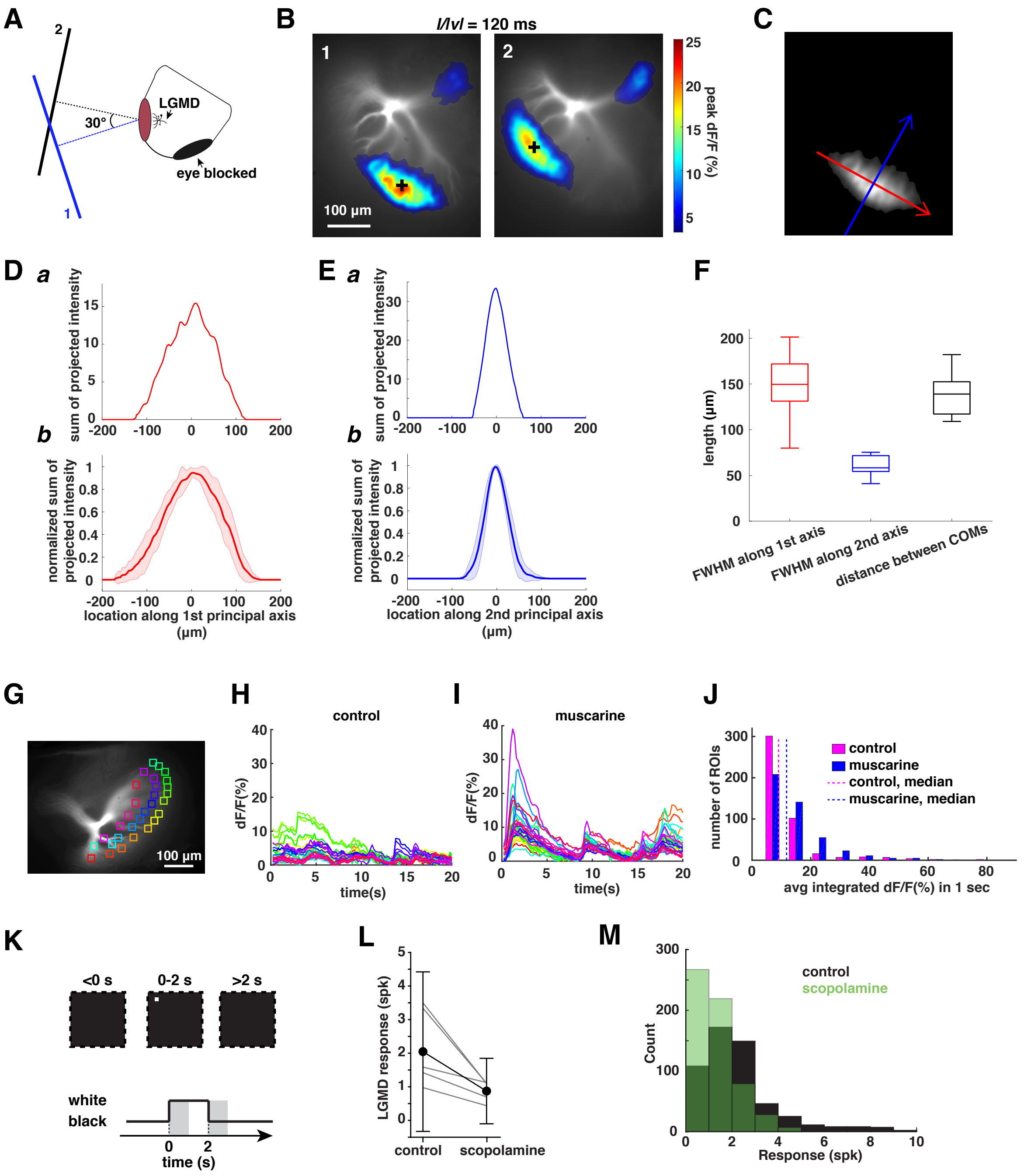

A
2
LGMD
30°
eye blocked
1
B
l/|v| = 120 ms
1
2
100 µm
peak dF/F (%)
C
D
a
b
sum of projected intensity
normalized sum of projected intensity
location along 1st principal axis (µm)
E
location along 2nd principal axis (µm)
F
length (µm)
FWHM along 1st axis
FWHM along 2nd axis
distance between COMs
G
100 µm
H
control
dF/F(%)
time(s)
I
muscarine
J
number of ROIs
control
muscarine
control, median
muscarine, median
avg integrated dF/F(%) in 1 sec
K
<0 s
0-2 s
>2 s
white
black
time (s)
L
LGMD response (spk)
control
scopolamine
M
Count
control
scopolamine
Response (spk)

Figure S4

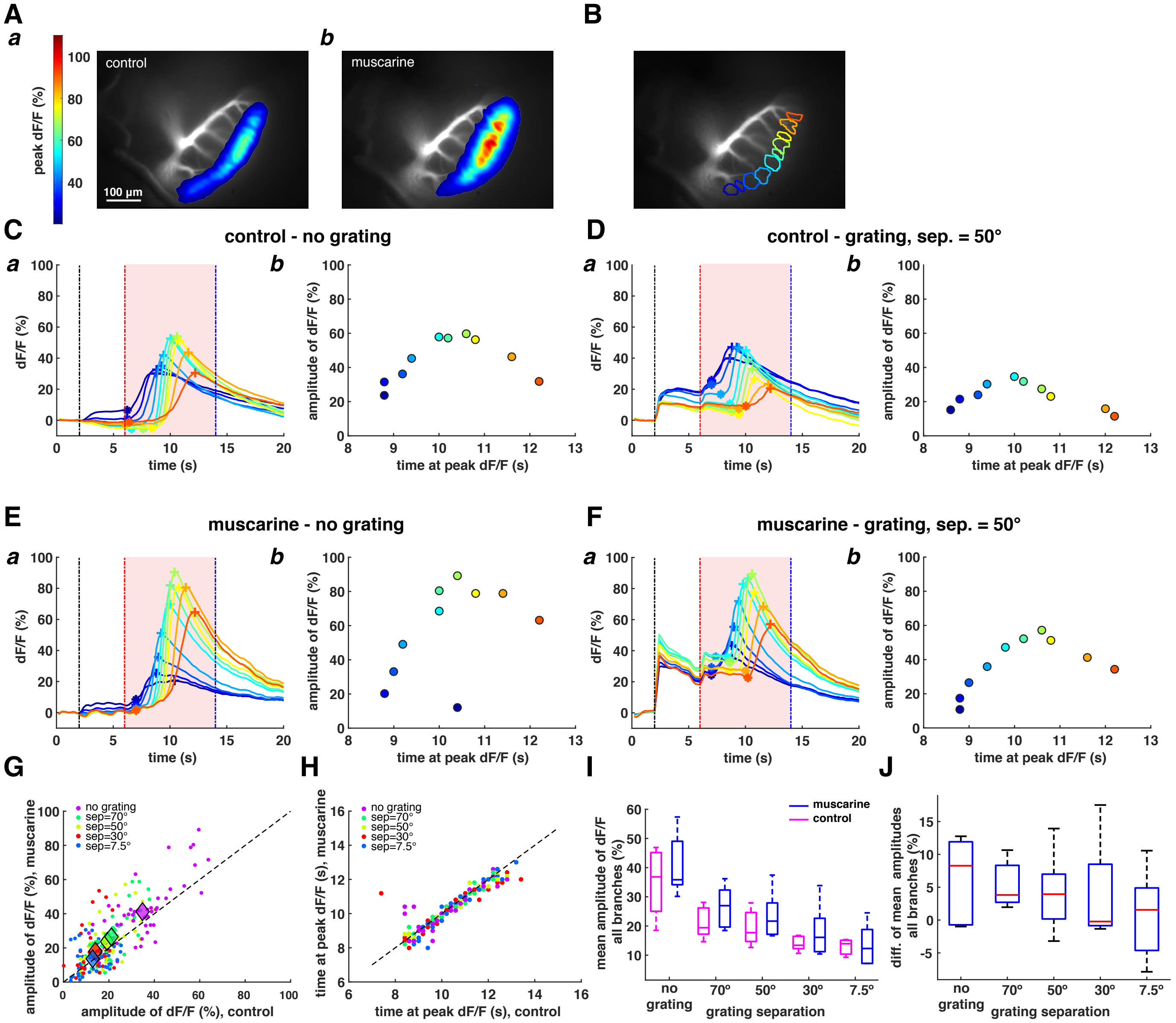
A
a
b
B
peak dF/F (%)
control
muscarine
100 µm
C
control - no grating
D
control - grating, sep. = 50°
dF/F (%)
time (s)
amplitude of dF/F (%)
time at peak dF/F (s)
E
muscarine - no grating
F
muscarine - grating, sep. = 50°
G
H
I
J
no grating
sep=70°
sep=50°
sep=30°
sep=7.5°
amplitude of dF/F (%), muscarine
amplitude of dF/F (%), control
time at peak dF/F (s), muscarine
time at peak dF/F (s), control
muscarine
control
mean amplitude of dF/F all branches (%)
diff. of mean amplitudes all branches (%)
no grating
70°
50°
30°
7.5°
grating separation

Figure S5

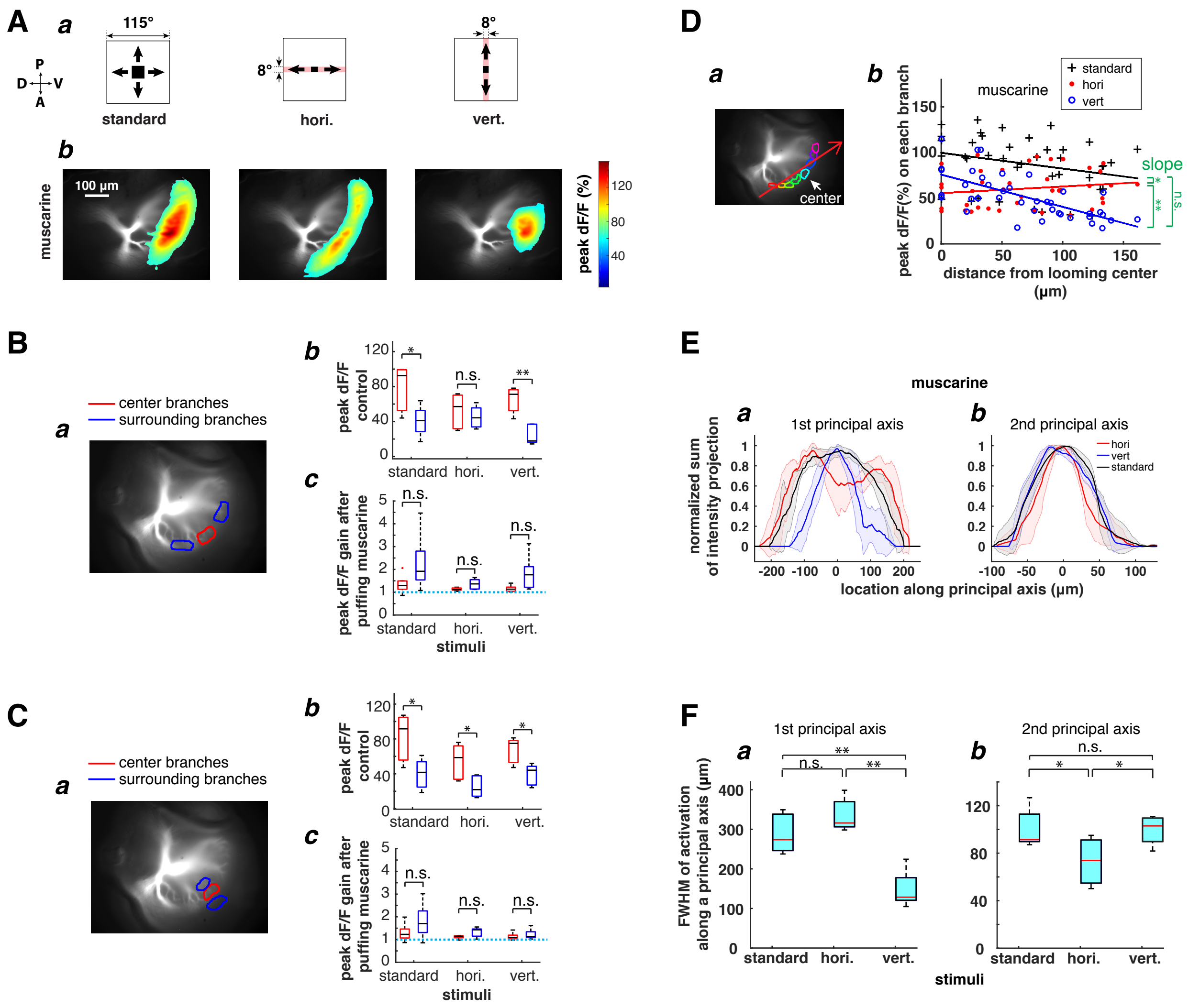

A a
115°
8°
8°
P
D
V
A
standard
hori.
vert.
b
100 µm
muscarine
peak dF/F (%)
120
80
40
B a
center branches
surrounding branches
b
peak dF/F control
c
peak dF/F gain after puffing muscarine
n.s.
standard
hori.
vert.
stimuli
C a
center branches
surrounding branches
D a
center
b
muscarine
standard
hori
vert
peak dF/F(%) on each branch
distance from looming center (µm)
slope
E
muscarine
1st principal axis
2nd principal axis
normalized sum of intensity projection
location along principal axis (µm)
hori
vert
standard
F
1st principal axis
2nd principal axis
FWHM of activation along a principal axis (µm)
stimuli

Figure S6

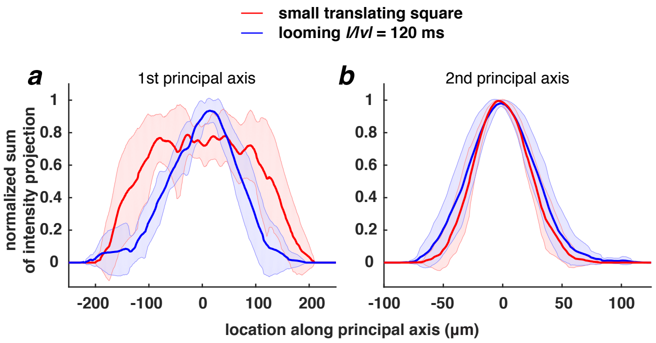

small translating square
looming l/|v| = 120 ms
a
1st principal axis
b
2nd principal axis
normalized sum of intensity projection
1
0.8
0.6
0.4
0.2
0
-200
-100
0
100
200
-100
-50
0
50
100
location along principal axis (μm)

Figure S1. Additional properties of calcium responses to small square and looming stimuli. Related to Figure 1.

(A) Response to a small square stimulus. Representative dF/F change elicited in a single dendritic branch by a 50 ms long, 8x8º square off flash (mean and sd of 5 trials in one animal). The red line indicates the timing of the stimulus.

(B) Timing of peak dF/F relative to stimulus peak time as a function of distance from dendritic looming center. Box plots of the distance from looming center at -0.22, -0.02, 0.18 s relative to stimulus peak time (**$p<0.01$, 2-sample t-test). Data pooled from 7 animals (58 dendritic branches).

Figure S2. Time course of calcium responses measured with the dyes OGB-1 or OGB-5N to looming stimuli of different size to speed ratios. Related to Figure 2.

(A) ***a***: Excitatory dendritic field of the LGMD with selected dendritic branches indicated by different colors. ***b***: time course of looming stimulus angular size. ***c***: relative fluorescence change recorded with OGB-1. Each trace is colored to match the dendrite colors on left and is an average of 3 trials in one animal. The right inset is a zoomed-in view of the same traces.

(B) Same as (A), but for OGB-5N in a different animal.

(C) Excitatory dendritic field of the LGMD with selected dendritic branches indicated by different colors. White dashed lines indicated the location of the intracellular sharp electrode.

(D) ***a***: time course of the angular size of the looming stimulus with $l/|v| = 30$ ms. ***b***: membrane potential (V_m) in the LGMD excitatory dendrite is shown in black solid trace. Gray shading, standard deviation of 3 trials. Thin solid colored traces are the time courses of the average dF/F within the selected dendritic branches in (C). The inset is a zoomed-in view of the same traces. In (D-I) the blue dashed line is the start of the looming stimulus and the red dashed line is the time of maximal stimulus size. Projected collision would occur 20 ms after maximal stimulus size.

(E) Same analysis as in (D) but in response to a looming stimulus with $l/|v| = 60$ ms. Projected collision would occur 40 ms after maximal stimulus size.

(F) Same analysis as in (D) but in response to a looming stimulus with $l/|v| = 120$ ms. Projected collision would occur 80 ms after maximal stimulus size.

(G) Firing rate of the LGMD in response to a looming stimulus with $l/|v| = 30$ ms. Blue shading, standard deviation of 5 animals.

(H) Firing rate of the LGMD in response to a looming stimulus with $l/|v| = 60$ ms. Blue shading, standard deviation of 5 animals.

(I) Firing rate of the LGMD in response to a looming stimulus with $l/|v| = 120$ ms. Blue shading, standard deviation of 5 animals.

Figure S3. Analysis and quantification of calcium responses on the excitatory dendritic field of the LGMD in various experimental conditions. Related to Figure 3.

(A) Schematics of two display positions in front of the locust eye with an angle difference of 30°. The ipsilateral LGMD neuron is depicted schematically (black arrow). The other eye is blocked by wax.

(B) Activation on the excitatory dendritic field in response to looming stimuli ($l/|v| = 120$ ms) simulating approach from two different angles, as in (A), is shown in the left and right panels, respectively. Each black cross indicates the center-of-mass of the activation on the excitatory dendritic field.

(C) The two principal axes of the activated area on the excitatory dendritic field for the data shown on the left panel in (B). The red and blue arrows indicate the 1st and 2nd principal axis, respectively.

(D) ***a***: sum of peak dF/F projected onto the first principal axis and plotted as a function of the projection location along the first principal axis (n = 1 animal, 1 looming angle/animal). ***b***: same analysis as ***a*** but after normalization to the peak of the curve for each animal across animals. Thick line and red shading indicate the mean and the standard deviation (n = 5 animals, 2 looming angles/animal).

(E) ***a***: sum of peak dF/F projected onto the second principal axis and plotted as a function of the projection location along the second principal axis (n = 1 animal, 1 looming angle/animal). ***b***: same analysis as ***a*** but after normalization to the peak of the curve for each animal across animals. Thick line and blue shading indicate the mean and the standard deviation (n = 5 animals, 2 looming angles/animal).

(F) Comparison of the FWHM of the curve in (D) ***b*** and (E) ***b*** with the distance between the center-of-masses (COMs) in response to looming stimuli on displays with 30° in orientation difference (n=5 animals).

(G) 30 selected region-of-interests (ROIs) in an example LGMD neuron.
(H) One example trial of dF/F (%) in each selected ROIs in (G) before puffing muscarine.
(I) One example trial of dF/F (%) in each selected ROIs in (G) after puffing muscarine.
(J) Histogram of the average integrated dF/F per second in a 20 second recording for control and after puffing muscarine without visual stimuli. Dotted magenta and blue lines are the median of the average integrated dF/F per second for control and muscarine, respectively (n=5 animals, 3 trials per animal, 30 ROIs per trial, $p=4.7 \cdot 10^{-5}$, Wilcoxon sign-rank test).
(K) Schematic of the small flash stimuli. 1x1° and 2x2° white squares were presented on a black monitor at time 0 s. After 2 seconds the squares disappeared. The number of spikes recorded extracellularly during the second after onset and offset (gray shaded regions at bottom) were counted as the LGMD response. Squares of both sizes were presented at 25 screen locations.
(L) For each animal, the response to small flashes decreased after puffing scopolamine ($p \leq 0.001$, Wilcoxon rank sum test, n=5). Grey lines show mean data from individual animals. Black lines and dots are the mean response with error bars of ±1 sd (corresponding to the spread of the distributions illustrated in M). Reduction in response occurred for both the 'on' and 'off' flash for the 1 and 2° stimuli, so data was pooled.
(M) Spike count histograms of the two conditions show the reduction in response after scopolamine application.

Figure S4. Test of the influence of muscarine on lateral inhibition. Related to Figure 4.
(A) ***a***: activation on the LGMD excitatory dendritic field in response to the small translating visual stimulus. ***b***: activation on the LGMD excitatory dendritic field in response to the small translating visual stimulus after puffing muscarine (n=5). Note increased activation relative to control.
(B) Excitatory dendritic field of the LGMD with selected dendritic branches indicated by different colors.
(C) ***a***: time courses of the average dF/F at each of the selected dendritic branches in (B), with matched colors, in response to a small translating visual stimulus as in Fig. 4A. Each trace is an average of dF/F over 3 trials. Black vertical dashed line: time when the small visual stimulus appears. Red vertical dashed line: time when the small visual stimulus starts translating. Blue vertical dashed line: time when the small visual stimulus moves completely outside of the display. The asterisk and plus markers on each curve indicate the baseline and the peak dF/F of the calcium response to the translating stimulus, respectively. ***b***: For each dendritic branch the amplitude of dF/F is computed by subtracting the baseline (indicated by the asterisk marker in ***a***) from the peak dF/F (indicated by the plus marker in ***a***) for each curve in ***a*** with matched color and plotted as a function of the time at peak dF/F.
(D) ***a***: time courses of the average dF/F (over 3 trials) at each of the selected dendritic branches in (B), with matched colors, in response to the small translating visual stimulus with lateral drifting gratings (with a separation of 50°) as in Fig. 4B. Black vertical dashed line: time when the small visual stimulus and lateral gratings appear. Red vertical dashed line: time when the lateral gratings starts drifting and 0.2 s later the small visual stimulus starts translating. Blue vertical dashed line: time when the small visual stimulus moves completely outside of the display and the lateral gratings stop moving. The asterisk and plus markers on each curve indicate the baseline and the peak dF/F of the calcium response to the translating stimulus, respectively. ***b***: plot similar as in (C) ***b***. Note decreased dF/F relative to (C).
(E) Same visual stimuli and plotting conventions as in (C), after puffing muscarine. Note increased dF/F relative to (C).
(F) Same visual stimuli and plotting conventions as in (D), after puffing muscarine. Note decreased dF/F relative to (E), suggesting lateral inhibition is unaffected by mAChRs.
(G) The amplitude of dF/F on every selected dendritic branch after puffing muscarine versus before puffing (5 animals, 47 dendritic branches total, average of 3 trials per condition). Purple dots: small translating stimuli with no drifting lateral gratings. Green, light green, red, and blue dots: small translating stimuli with drifting lateral gratings separated by 70°, 50°, 30° and 7.5°, respectively. Purple, green, light green, red and blue diamonds are the mean (center-of-mass) of the purple, green, light green, red and blue dots, respectively. No change in dF/F was observed after puffing muscarine.
(H) Time at peak dF/F on every selected dendritic branch after versus before puffing muscarine. Same plotting conventions as in (G). No change in peak timing was observed.
(I) Box plots of the mean amplitudes of dF/F for all the branches in each animal in response to small translating stimuli with no drifting lateral gratings, and with gratings separated by 70°, 50°, 30° and 7.5°, respectively. Blue, after puffing muscarine. Magenta, before puffing muscarine (n=5 animals; p=0.0004 for control and p=0.001 for muscarine, one-way ANOVA; p=0.07 for control vs. muscarine, two-way ANOVA). Note decreased dF/F with grating separation, consistent with lateral inhibition.

(J) Box plots of the difference in mean peak amplitudes for all branches in each animal after puffing muscarine and before puffing, in response to small translating stimuli with no drifting lateral gratings, and with gratings of separations 70°, 50°, 30° and 7.5°, respectively. No significant statistical difference of the means was found between any two groups (n=5 animals, p=0.72, one-way ANOVA). Thus muscarine did not affect lateral inhibition.

Figure S5. Calcium responses to looming stimuli restricted to horizontal and vertical bands after puffing muscarine. Related to Figure 5.

(A) ***a***: from left to right, schematics of a standard looming stimulus, a looming stimulus restricted in a horizontal band (along the dorsal-ventral eye axis) and in a vertical band (along the posterior-anterior eye axis), respectively. ***b***: from left to right, activation on the LGMD excitatory dendrite in response to the same stimuli after puffing muscarine. Same neuron as in Fig. 5A. Note increased dF/F for all stimuli.

(B) ***a***: three selected ROIs on different dendritic branches. The center region represented in red mapped to the looming center. The two blue regions are approximately symmetrically placed on either side of the center region along the 1st principal axis. ***b***: peak dF/F in the center or surrounding ROIs for standard, horizontal and vertical band-restricted looming before puffing muscarine. ***c:*** The ratio of mean peak dF/F of the center branches (red, corresponding to the region selected in red in ***a***) and surrounding branches (blue, corresponding to the region selected in blue in ***a***) after puffing muscarine to that before puffing in response to the standard looming stimulus, the looming stimulus restricted in a horizontal band, or restricted in a vertical band, respectively. (n=5 animals, *p<0.05, n.s.: not significant, i.e., p>0.05, two-sample t-test assuming unequal variances). Note increased responses, but unchanged gain between center and surrounding regions for all stimuli.

(C) Same analyses as in (B) except for the selection of two regions (in blue) approximately symmetrically placed on either side of the center region (in red) along the same dendritic branch (corresponding approximately to 2nd principal axis). Note increased dF/F for all stimuli (***b***), but unchanged gain (***c***).

(D) ***a***: 8 selected regions-of-interest (ROIs, same as in Fig. 5B) aligned with 1st principal axis are represented by different colors in an example LGMD neuron. White arrow points to the dendritic region that maps to the looming center. Red arrows indicate the 1st principal axis of the activated area during the looming response. ***b***: peak dF/F in the 8 selected ROIs pooled across 5 animals in response to the standard looming stimulus (black crosses), the looming stimulus restricted in a horizontal band (red dots) or restricted in a vertical band (blue circles) after puffing muscarine. Solid lines are the linear regression of the data (n=5, *p<0.05, **p<0.01, ANCOVA test of slopes).

(E) Sum of peak dF/F projected onto the 1st (***a***) and 2nd (***b***) principal axes and plotted as a function of the projection location along that principal axis after normalizing to the peak of the curve in response to the standard looming stimulus (black), the looming stimulus restricted in a horizontal band (red) or restricted in a vertical band (blue) after puffing muscarine. Red, black and blue shadings, standard deviation of 5 animals. Note increase relative to control (Fig. 5C) and similarity of red and black lines in ***a*** and of blue and black lines in ***b***.

(F) Box plots of the full-width at half-maximum (FWHM) of the curves in (E) along the first principal axis (***a***) and the second principal axis (***b***) in response to the standard or the horizontal/vertical band-restricted looming stimulus after puffing muscarine. The FWHM after puffing muscarine is significantly higher than that before puffing muscarine except for the looming stimulus restricted in a vertical band along the 2nd principal axis (n=5 animals, *p<0.05, **p<0.01, paired t-test, n.s., not significant, i.e., p>0.05). Note similarity of standard and hori. along 1st principal axis and of standard and vert. along 2nd principal axis, suggesting saturation of the responses after muscarine.

Figure S6. Comparison of dendritic activation area for small translating squares and looming stimuli. Related to Figure 5.

Sum of peak dF/F projected onto the 1st (***a***) and 2nd (***b***) principal axes and plotted as a function of the projection location along that principal axis after normalizing to the peak of the curve in response to the standard looming stimulus (blue), or a small translating square (red; same as stimuli in Fig. 4A). Solid lines are mean and shaded areas, standard deviations over 10 animals. The FWHM of the two stimuli along 1st principal axis are significantly different ($p=8.37\cdot 10^{-5}$), but those along the 2nd principal axis are not (p=0.14; two-sample t-test).
